# Binding of N-methylscopolamine to the extracellular domain of muscarinic acetylcholine receptors

**DOI:** 10.1038/srep40381

**Published:** 2017-01-16

**Authors:** Jan Jakubík, Alena Randáková, Pavel Zimčík, Esam E. El-Fakahany, Vladimír Doležal

**Affiliations:** 1 Institute of Physiology, Czech Academy of Sciences, 14220 Prague, Czech Republic; 2 Department of Experimental and Clinical Pharmacology, University of Minnesota College of Pharmacy, Minneapolis, MN 55455, USA (EEE)

## Abstract

Interaction of orthosteric ligands with extracellular domain was described at several aminergic G protein-coupled receptors, including muscarinic acetylcholine receptors. The orthosteric antagonists quinuclidinyl benzilate (QNB) and N-methylscopolamine (NMS) bind to the binding pocket of the muscarinic acetylcholine receptor formed by transmembrane α-helices. We show that high concentrations of either QNB or NMS slow down dissociation of their radiolabeled species from all five subtypes of muscarinic acetylcholine receptors, suggesting allosteric binding. The affinity of NMS at the allosteric site is in the micromolar range for all receptor subtypes. Using molecular modelling of the M_2_ receptor we found that E172 and E175 in the second extracellular loop and N419 in the third extracellular loop are involved in allosteric binding of NMS. Mutation of these amino acids to alanine decreased affinity of NMS for the allosteric binding site confirming results of molecular modelling. The allosteric binding site of NMS overlaps with the binding site of some allosteric, ectopic and bitopic ligands. Understanding of interactions of NMS at the allosteric binding site is essential for correct analysis of binding and action of these ligands.

Five subtypes of muscarinic receptors have been cloned^1^. Muscarinic receptors are expressed throughout the mammalian body where they regulate many physiological functions that are specific to different receptor subtypes^2^. Therefore, muscarinic receptors are potential targets for therapeutic intervention in various pathological conditions, most importantly in neurodegenerative disorders^3^,^4^. Treatment of such disorders without significant undesired effects necessitates using ligands that are selective for certain muscarinic receptor subtypes. However, the orthosteric binding site of the muscarinic receptor is structurally conserved among receptor subtypes^5^,^6^. This makes it difficult to attain subtype selectivity of ligands that bind to the primary site. Therefore, attention of researchers has turned to ligands that interact with the extracellular domain of the muscarinic receptor that differs among receptor subtypes. The extracellular domain of muscarinic receptors has been shown to bind a wide variety of selective ligands including allosteric modulators^7^,^8^,^9^,^10^,^11^,^12^,^13^,^14^, ectopic ligands^15^ and bitopic ligands^16^,^17^,^18^.

Although the crystal structure of muscarinic receptors shows a single binding site for orthosteric ligands^5^,^6^,^19^,^20^, deviations from single-step bimolecular reaction were observed in binding of the orthosteric antagonist quinuclidinyl benzilate (QNB). Receptor isomerization upon QNB binding and the possibility of simultaneous binding of two molecules of QNB were proposed to explain this phenomenon^21^,^22^. Subsequently, a tandem two-site model for ligand binding was proposed^23^. This model suggests that orthosteric ligands bind transiently to the allosteric site on the muscarinic receptor (located in the extracellular domain) both on their way to and from the orthosteric site (located in the binding pocket among transmembrane α-helices). The tandem two-site model was confirmed experimentally^24^ as well as *in silico* by simulation of molecular dynamics of tiotropium binding^6^. Additional lines of evidence for interaction of orthosteric ligands with extracellular loops (ECL) include, for example, mutations in the second extracellular loop (ECL2) of the M_1_ receptor that lowered affinity of the receptor for QNB and decreased the potency of acetylcholine^25^ and mutations in the ECL2 of the M_3_ receptor that lowered affinity of the receptor for both the antagonist N-methylscopolamine (NMS) and the agonist carbachol^26^. Interaction of orthosteric ligands with extracellular domains was also described at other aminergic GPCRs, e.g. β-adrenergic, adenosine^27^,^28^,^29^ suggesting common molecular mechanism of binding in this sub-class of GPCRs.

In the present study we probed deeply into the mechanisms and kinetics of binding of the orthosteric antagonist NMS to the muscarinic receptor using site-directed mutagenesis and molecular modelling. We identified amino acids at the vestibule (Fig. 1) of the binding pocket between ECL2 and ECL3 with which NMS specifically interacts in an allosteric manner. This finding complicates interpretation of experiments in which radiolabeled antagonists such as NMS and QNB that are presumed to interact exclusively with the receptor orthosteric domain are used as tracers in studying of binding properties of other ligands that are not available in a radioactive form.

## Results

### Binding properties of wild type muscarinic receptors

The classical tritiated antagonists N-methylscopolamine ([^3^H]NMS) and quinuclidinyl benzilate ([^3^H]QNB) bound to individual subtypes of muscarinic receptors with similar affinity, that was slightly lower at M_2_ receptors (Table 1). Dissociation of these two radioligands was faster at the M_2_ than at the remaining subtypes, while dissociation of [^3^H]NMS from M_5_ receptors was substantially slower than from others. At concentrations of 100 μM and higher, NMS concentration-dependently slowed down dissociation of [^3^H]NMS from all subtypes of muscarinic receptors (Fig. 2, left). The observed dissociation rates (k_obs_) were plotted against the concentration of cold NMS. The apparent affinity of NMS for the receptor-[^3^H]NMS complex (K_A_) was calculated according to Lazareno and Birdsall (1995) by fitting Eq. 4 to data. In case of biphasic dissociation (Eq. 3 fitted better to the data than Eq. 2) value of the slower phase was used. The decrease in dissociation rate in relation to the concentration of cold NMS was steep in case of M_2_ and M_5_ receptors, necessitating introduction of a slope factor nH to Eq. 4. Calculated nH was 1.5 in both cases. Calculated K_A_ of NMS was in the micromolar range for all subtypes and was highest (lowest apparent affinity) at M_3_ and lowest (highest apparent affinity) at M_5_ receptors (Table 1). Similar apparent affinities of NMS for the allosteric site were found by measurement of the effects of various concentrations of NMS on [^3^H]QNB dissociation (Supplementary data, Figure SI2, Table SI1).

Also QNB concentration-dependently slowed down dissociation of [^3^H]QNB from all subtypes of muscarinic receptors (Fig. 3, left). However, solubility of QNB in water is limited and good solvents of QNB such as DMSO and acetic acid substantially affected [^3^H]QNB dissociation. The highest tested concentration of QNB was thus limited to 1 mM. Observed k_off_ of [^3^H]QNB in the presence of 1 mM cold QNB is listed in Table 1. Inhibition of [^3^H]QNB dissociation by 1 mM QNB was not complete and K_A_ of QNB could not be reliably estimated (Fig. 3, right).

### Binding properties of mutant M_3_ receptor

**Figure 1:**
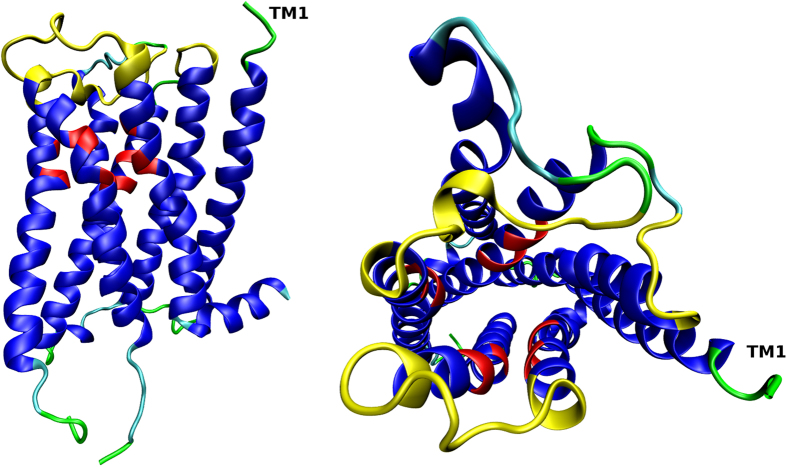
M_2_ muscarinic receptor. Vestibule (yellow) to the binding pocket with the orthosteric site (red) of the M_2_ receptor as viewed from side (left, extracellular side up) and from the extracellular side (right). Structures: blue – α-helix, cyan – coil; green – turn.

The vestibule (Fig. 1) to the binding pocket is formed mainly by the second (ECL2) and the third (ECL3) extracellular loops. Because ECL3 is 3-times shorter than ECL2 it was mutated first. The M_3_ receptor that has highest K_A_ for NMS was mutated in the ECL3 to corresponding amino acids of the M_2_ receptor that has the second lowest K_A_, with the aim to decrease the K_A_. Mutations of the M_3_ receptor in the ECL3 affected the affinity for [^3^H]NMS as well as binding kinetics and apparent affinity of the receptor-[^3^H]NMS complex for NMS (Fig. 4, Table 2). Single amino acid mutations S519P and K523N did not affect the affinity of [^3^H]NMS but accelerated its dissociation. Double mutation of these two amino acids (SK- > PN) also did not change the affinity of [^3^H]NMS but further accelerated its dissociation. All other tested combinations of mutations in the ECL3 of the M_3_ receptor lowered the affinity for [^3^H]NMS and accelerated its dissociation to the rate observed at M_2_ receptors.

Except for mutation S519P, all tested mutations in the ECL3 of M_3_ receptor increased the apparent affinity (lowered K_A_) of the receptor-[^3^H]NMS complex for NMS. Major contribution to the decrease in K_A_ was by mutation K523N. Double mutation of these two amino acids (SK- > PN, see Methods, Residue naming and numbering) had the same K_A_ as single mutation K523N. All M_3_ mutants containing KFN mutation in the ECL3 had K_A_ as low as the wild type M_2_ receptor. Therefore reciprocal mutations in the ECL2 have not been done.

### Molecular dynamics

Free molecular dynamics (MD) of QNB in the complex with the M_2_ or M_3_ receptor in membrane/water environment was simulated. Energetic analysis by Simulation Quality Analysis tools showed that the simulated system is stable and that QNB remains bound at the orthosteric binding site. Calculation of root mean square fluctuations (RMSF) of individual amino acids showed that RMSF of the backbone as well as heavy atoms of the ECL2 and the ECL3 are greater at the M_2_ receptor than at the M_3_ receptor (Fig. 5) demonstrating higher flexibility of the vestibule of the binding pocket at the M_2_ than at the M_3_ receptor. Also the RMSF of the M_2_ receptor was higher in some parts of transmembrane (TM) helix 5 and 6. MD of M_2_ and M_3_ receptors in complex with NMS or tiotropium was also simulated and yielded the same RMSF profiles.

Analysis of intramolecular interactions over MD trajectory in Simulation Interaction Diagram tool showed that conformation of the ECL2 of the M_3_ receptor is largely stabilised by interactions of two conserved amino acids. Conserved D142 (D^3.26^ according Ballesteros Weinstein numbering^30^ see Methods, Residue naming and numbering) in the extracellular edge of TM 3 forms hydrogen bonds with conserved R213 in the ECL2. Conserved E219 (E^4.99^) in the middle of ECL2 interacts via hydrogen bonds, ionic interactions and water bridges with K522 (K^7.32^) in the ECL3 and R132 in the ECL1 (Fig. 6). The former stabilising interaction is also present at the M_2_ receptor (D97 hydrogen bonding to R169), however, the later ones are absent from the M_2_ receptor where conserved E175 (E^4.99^) interacts weakly with Y83 in the extracellular edge of TM 2. Glutamate E172 (E^4.96^) forms a hydrogen bond with the backbone nitrogen of E175 but this hydrogen bond does not have a stabilising effect on ECL2 flexibility (Fig. 6). It should be noted that a hydrogen bond between E219 (E^4.99^) and K522 (K^7.32^) is not observed in the crystal structure of the M_3_ receptor. This hydrogen bond is readily formed during MD, where it is present with cca 60% occupancy at cca 35% of time frames. At additional 15% of time frames E219 interacts with K522 via ionic interaction.

Comparison of free MD trajectory of the wild type M_2_ receptor with trajectory of D97N and D97A mutants showed increased RMSF of the backbone as well as heavy atoms of the ECL2 and ECL3 of mutants (Fig. 7). The highest RMSF in the ECL2 was found for R169 and the highest RMSF in the ECL3 loop was found for I409. Analysis of intramolecular interactions over MD trajectory in Simulation Interaction Diagram tool showed that in the wild type M_2_ receptor R169 interacts via hydrogen bonds with D97 (Fig. 6). This interaction is missing at the D97N as well as D97A mutants. The increase in RMSF of the ECL2 is thus due to these missing hydrogen bonds. Neither I409 nor another residue in the ECL3 displays specific and frequent interactions with any receptor residue. However, the majority of sporadic interactions of the ECL3 occur with the ECL2. Thus the increase in RMSF of the ECL3 of mutants is due diminish in plethora of interactions between ECL2 and ECL3 that is due to increased flexibility of the ECL2.

### Steered molecular dynamics

*In silico*, dissociation of QNB and NMS was studied by simulation of MD steered dissociation. During MD acceleration inversely proportional to the distance between centre of ligand and centre of bottom of binding pocket in the direction from the bottom of binding pocket was applied to the ligand. Initial acceleration needed to initiate the dissociation was 3500 nm.ns^−2^ for QNB and 3000 nm.ns^−2^ for NMS (Fig. 8, left). At simulations with initial acceleration of 4000 nm.ns^−2^ both NMS and QNB remained in the vestibule of the binding pocket around 1.6 nm from bottom of the binding pocket with smaller but still positive binding energy (Fig. 8, left). Initial acceleration of 6000 or 8000 nm.ns^−2^ caused complete dissociation of NMS from the receptor (Fig. 8, bottom).

Initial acceleration of 4000 nm.ns^−2^ was used to test the effects of acceleration direction on ligand dissociation during steered MD (Fig. 9, Supplementary Videos 1 and 2). While QNB remained in the vestibule of the binding pocket during entire MD of steered dissociation (Fig. 9, left), the trajectory of NMS was much more prone to the acceleration direction (Fig. 9, right). The direction of acceleration affected steered dissociation in the both ways: either delayed departure from the orthosteric binding site or accelerated dissociation.

For comparison, MD of steered ligand dissociation from D97N mutant M_2_ receptor was simulated (Fig. 10, Supplementary Videos 3 and 4). Initial acceleration of 4000 nm.ns^−2^ was applied to the ligand at various directions. The departure of both QNB and NMS from the orthosteric binding site was almost instant. They remained in the vestibule of the binding pocket transiently and then started to fully dissociate from the receptor.

Trajectories of MD of steered QNB and NMS dissociation at wild type and D97N mutant M_2_ receptor were analysed to identify ligand-receptor interactions over the course of simulation. Frequency of hydrogen bonding and π-π interactions with individual residues is shown in Fig. 11. In the orthosteric binding site QNB interacts via hydrogen bonds primarily with N404 and via π-π interactions with Y104, W155, W400 and Y403. Hydrogen bonding of QNB with D103, S107 and Y403 is less abundant. With the exception of W155 and W400, interactions of QNB with amino acid residues in the orthosteric site are not affected by D97N mutation. In the orthosteric binding site NMS interacts via hydrogen bonds primarily with Y104 and N404 and via π-π interactions with Y104, W155, W400 and Y403. Hydrogen bonding of NMS with D103, and Y403 is less abundant. Similar to QNB, with the exception of W155 and W400, interactions of NMS with amino acid residues in the orthosteric site are not affected by the D97N mutation. In the vestibule of the binding pocket QNB interacts via hydrogen bonds primarily with E175 and N419 and via π-π interactions with W155, Y177, F181, W422 and Y426. Hydrogen bonding of QNB with E172, Y177, Q179 and S182 and π-π interaction with Y83 is less abundant. Mutation D97N reduced interaction of QNB via hydrogen bonds with E175 and N419 and also reduces interaction of QNB via π-π interaction with F181 and W422 but not with Y177. Similar to QNB, NMS interacts in the vestibule of the binding pocket via hydrogen bonds primarily with E175 and N419 (but also with E172) and via π-π interactions with Y177, while π-π interactions with F181, W422 and Y426 are less abundant. Mutation D97N primarily reduced interaction of NMS via hydrogen bonds with E172, E175 and N419 and also reduces interaction of NMS via π-π interaction with Y177.

Time-wise, upon application of acceleration to ligand (QNB or NMS) the hydrogen bond to D103 (D^3.32^) is readily disrupted while the hydrogen bond to N404 (N^6.52^) persists. In case of NMS the hydrogen bond to D103 is transiently replaced by cation-π interaction with neighbouring Y104. Then the nitrogen group turns towards the extracellular opening and contacts with W400 and W155 are lost. Following a delay the ligand starts to dissociate from the orthosteric site to the vestibule of the binding pocket where it interacts with N419 (Fig. 11, left). Before reaching the vestibule ligand may transiently interact with Y177, F181 or S182. The ligand translocates within the vestibule from N419 to E175 (Fig. 12, right) with a delay that depends on the magnitude and direction of acceleration applied to the ligand. During translocation from N419 to E175 NMS may interact with both residues concurrently and may form hydrogen bonds with the oxygen in the azatricycle group to N419 and with the hydroxyl group to E175 (Supplementary Information, Figure SI4). Concurrent interaction with both amino acids is not possible in case of QNB that can form hydrogen bond only by hydroxy group. While in the vestibule of the binding pocket QNB as well as NMS may transiently interact with other amino acids, namely E172, Y177, Q179 and S182 (Supplemental Information, Figures SI5 and SI6). Again, the ligand starts to fully dissociate from the receptor with a delay that depends on the magnitude and direction of acceleration applied to it.

During MD sodium ions interact in the extracellular part of the receptor mainly with E172, E175 and Y177 (occupancy up to 20%) and less frequently with Y80, Y83, D173 (occupancy less than 5%). After ligand translocation from the orthosteric site to the allosteric site ligand displaces sodium ions associated with ECL2. Up to two sodium atoms entered the orthosteric binding pocket after ligand translocation. No association of sodium ions with D^2.50^ was observed during steered MD.

### Modelling of second ligand binding to the M_2_ receptor

Docking of the second molecule of ligand (QNB or NMS) to the vestibule of the binding pocket of crystal structure-based M_2_ receptor was unsuccessful due to unfavourable conformation of the ECL2 and orientation of key amino acids (E172, E175 and N419). In the crystal structure (3UON) E175 and E172 are oriented out from the vestibule and N419 is oriented down towards the binding pocket. Binding energies of resulting poses were extremely low suggesting no binding at all. Reorientation of these residues using induced fit was also unsuccessful. Thus, the second molecule of the ligand was docked to the M_2_ receptor with occupied orthosteric binding site by simulation of MD of steered ligand association. First, system of ligand-receptor complex in membrane was relaxed by 10 ns simulation of free MD. Then the second molecule of the ligand was placed extracellularly about 26 Å from the bottom of the orthosteric binding site and acceleration (initial value of 500 nm.ns^−2^) towards the orthosteric site was applied to the ligand for 2.5 ns. Then 10 ns of free MD was run. During 10 ns free MD at wt receptor both QNB and NMS remained in the vestibule to the binding pocket and interacted with E172, E175, Y177 and N419 exerting good binding (positive binding energies) (Fig. 13, Supplementary Videos 5 and 6). On the other hand, during 10 ns free MD at D97N mutant both QNB and NMS completely dissociated from the receptor (Fig. 14, Supplementary Videos 7 and 8). Again, during MD of steered association, ligands made hydrogen bonds primarily with E172, E175 and N419 and π- π interactions with Y177, F181 and W422 (Fig. 15).

### Binding properties of mutant M_2_ receptor

Simulation of MD has shown an important role of D97 of the M_2_ receptor in the stability of the ECL2. Mutation D97N is known to affect kinetics of the M_2_ receptor^7^,^23^. To test predictions of MD and verify previous findings D97 was mutated to asparagine. To exclude a possible artefact of polarization D97 was also mutated to alanine. Steered MD has shown that NMS primarily interacts at the vestibule of the binding pocket with E172, E175 and N419 via hydrogen bonds and with Y177 via π-π interaction. Thus, these four amino acids were individually mutated to alanine with the aim to impair NMS binding to the vestibule of the binding pocket. Except for Y177A, all tested mutations decreased affinity (increased K_D_) of [^3^H]NMS, accelerated [^3^H]NMS dissociation, and decreased affinity (increased K_A_) of NMS for receptor-[^3^H]NMS complex. (Table 3, Fig. 16). Mutation N419A in the ECL3 had the strongest effect among tested mutants. It increased the K_D_ of [^3^H]NMS, accelerated [^3^H]NMS dissociation, and increased K_A_ of NMS about 3-times. Mutations E172A and E175A had the weakest effect. They increased K_D_ of [^3^H]NMS, accelerated [^3^H]NMS dissociation, and increased K_A_ of NMS only about one and half fold. Mutations D97N and D97A had the medium effect. They increased K_D_ of [^3^H]NMS and increased K_A_ of NMS about two and half fold and accelerated [^3^H]NMS dissociation about one and half fold.

## Discussion

The main finding of this work is the identification of amino acids in the allosteric site in the vestibule (Fig. 1) of the binding pocket of the M_2_ receptor to which the orthosteric antagonist N-methylscopolamine (NMS) binds on its way to and from the orthosteric binding site. In the ECL3 NMS binds to N419. In the ECL2 NMS binds to E172 and the conserved E175. The conserved D97 (D^3.26^; numbering according Ballesteros and Weinstein^30^, see Methods, Residue naming and numbering) has a structure-stabilizing role by conditioning proper binding of NMS in the vestibule.

Deviations from single-step bimolecular reactions were observed in very early research of radioligand binding to muscarinic receptors. Ligand-induced receptor isomerization and the possibility of simultaneous binding of two molecules of ligand to the receptor were proposed to explain these deviations^21^,^22^. Bürgeisser *et al*.^31^ hypothesised the possibility that biphasic dissociation of QNB from muscarinic receptors may not be due to receptor isomerization but is due to racemic composition of the ligand. However, Walbroeck *et al*.^32^ have shown that the affinity of (S)-QNB is 100-fold lower than the affinity of (R)-QNB. So binding of labelled (S)-QNB cannot be detected at commonly used concentrations of the racemic tracer. A tandem two-site model was proposed based on attenuation of radioligand dissociation in the presence of high concentrations of competitive antagonists^23^. The tandem two-site model was confirmed experimentally for several orthosteric agonists and antagonists^24^. Antagonist binding (energetically favourable conformation) to the vestibule of the binding pocket was also demonstrated in simulation of molecular dynamics (MD) of tiotropium binding^6^. Interaction of orthosteric ligands with the allosteric site was also illustrated at β-adrenergic and adenosine receptors suggesting that tandem two-site mechanism of orthosteric ligand biding is common among aminergic GPCRs^27^,^28^,^29^.

**Figure 2:**
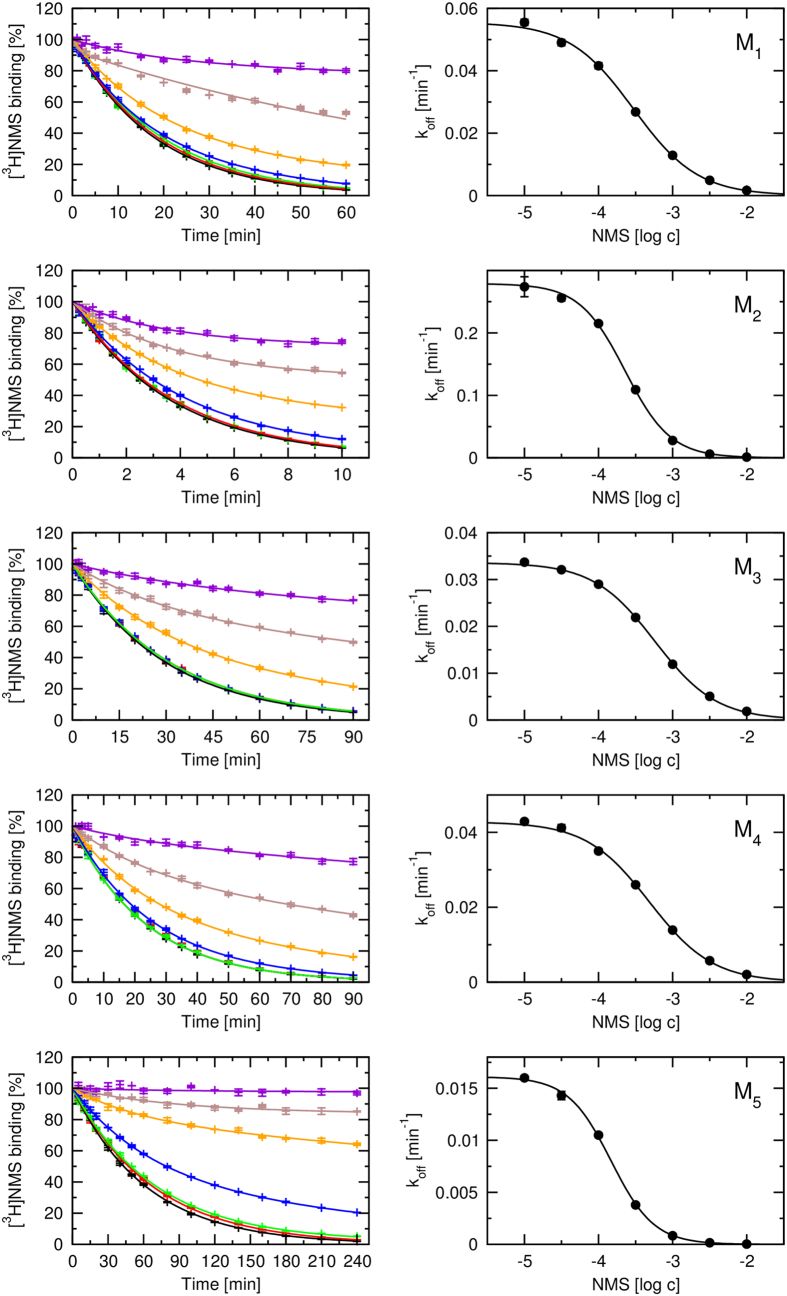
Dissociation of [^3^H]NMS. Time course of dissociation of 1 nM [^3^H]NMS from individual receptor subtypes (left) initiated by the addition of NMS at final concentrations varying from 10 μM (black) to 10 mM (violet) was followed for the time points indicated on the abscissa. Binding of [^3^H]NMS (ordinate) is expressed as per cent of binding at the beginning of dissociation. Dissociation rates (k_off_) calculated from time-courses according to Eq. 2 or Eq. 3 are plotted against the concentration of cold NMS used to initiate [^3^H]NMS dissociation (right). Eq. 4 or Eq. 5 was fitted to data to estimate K_A_ (Table 1). Data are means ± S.E.M. from 4 independent experiments performed in duplicates.

**Figure 3:**
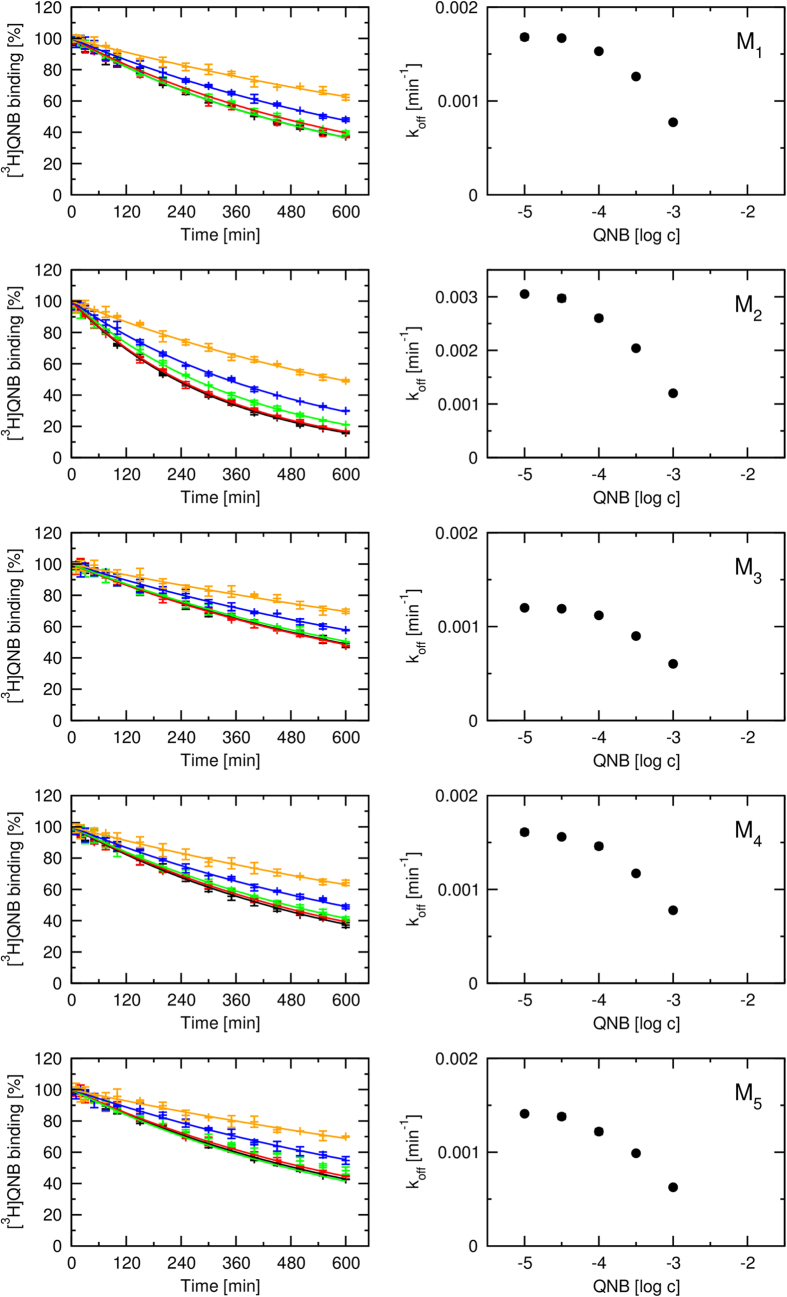
Dissociation of [^3^H]QNB. Time course of dissociation of 500 pM [^3^H]QNB from individual receptor subtypes (left) initiated by the addition of QNB at final concentrations varying from 10 μM (black) to 1 mM (yellow) was followed for the time periods indicated on the abscissa. Binding of [^3^H]QNB (ordinate) is expressed as per cent of binding at the beginning of dissociation. Dissociation rates (k_off_) calculated from time-courses according to Eq. 2 are plotted against the concentration of cold QNB used to initiate [^3^H]QNB dissociation (right). Data are means ± S.E.M. from 4 independent experiments performed in duplicates.

In the present study both unlabelled QNB and NMS slowed down dissociation of the radiolabeled tracer ([^3^H]QNB or [^3^H]NMS) from the orthosteric site in a concentration-dependent manner at all subtypes of muscarinic receptors (Figs 2 and 3, Supplementary data Figure SI2). Any alteration of ligand kinetics already maximized by a saturating concentration of a competitive antagonist is a hallmark of allosteric interactions. Estimation of the apparent affinity of NMS to the allosteric site at receptor-[^3^H]tracer complex according to Lazareno and Birdsall (1995)^33^ (Eq. 4) showed that NMS has the highest affinity for the allosteric site at the M_5_ receptor, second highest at the M_2_ receptor and lowest at the M_3_ receptor (Table 1). Importantly, the apparent affinities of NMS for the allosteric site determined by [^3^H]QNB dissociation were the same as those determined by [^3^H]NMS dissociation (Supplementary data, Figure SI2, Table SI1). Slow down of [^3^H]NMS dissociation by the orthosteric agonist carbachol was also reported^23^. These observations rule out kinetic artefacts. Similarity of apparent affinities of NMS and QNB to the extracellular site suggests that the strong negative cooperativity loosens ligand receptor interactions that ligand makes only a single interaction with the receptor at a time. Therefore subtle differences in conformation of the binding site (that would otherwise prevent multiple concurrent interactions) are indifferent to ligand binding. Because both ligands are similar in size and chemical properties, they may interact with the same amino acids by the same set of interactions and thus their apparent affinities are similar.

**Figure 4:**
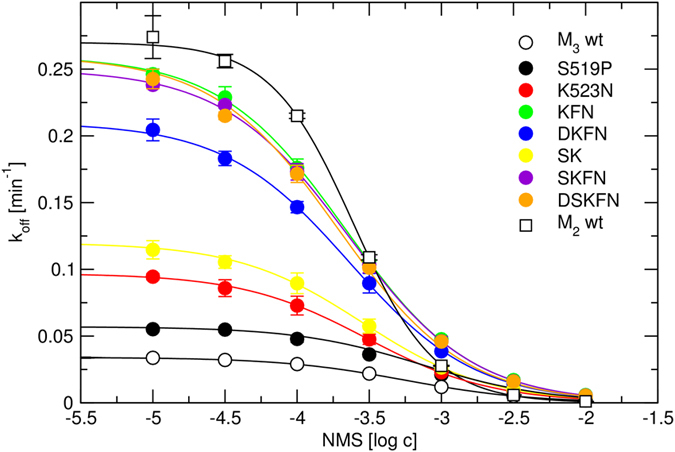
Dissociation rate constants of [^3^H]NMS from mutated M_3_ receptors. Dissociation rate constants (k_off_) of [^3^H]NMS from mutated M_3_ receptors calculated from time-courses according to Eq. 2 or Eq. 3 are plotted against the concentration of cold NMS used to initiate [^3^H]NMS dissociation. Eq. 4 was fitted to data to estimate K_A_ (Table 2). Data are means ± S.E.M. from 4 independent experiments performed in duplicates.

Extremely slow kinetics at the M_5_ receptors rendered kinetic experiments at this receptor subtype infeasible. On the other hand kinetics of the M_2_ receptor are the fastest among subtypes and quick equilibration is favourable for kinetic experiments. Moreover, at the time we started our study only the crystal structures of the M_2_ and M_3_ receptors^5^,^6^,^19^ (needed for modelling part) were available. Therefore, the M_3_ receptor was mutated in the ECL3 to the M_2_ sequence with the aim to increase the affinity of NMS for the receptor-[^3^HNMS] complex. Mutations in the ECL3 increased apparent affinity of NMS for the allosteric site on the M_3_ receptor (Fig. 4, Table 2). The major contribution to the increase in affinity of NMS for the allosteric site and to changes in the kinetics of [^3^H]NMS was brought about by mutation of lysine 523 to asparagine, pointing out the importance of the corresponding asparagine 419 of the M_2_ receptor in binding of NMS to the allosteric site. These mutations also affected the kinetics of [^3^H]NMS and its affinity for the orthosteric site.

**Figure 5:**
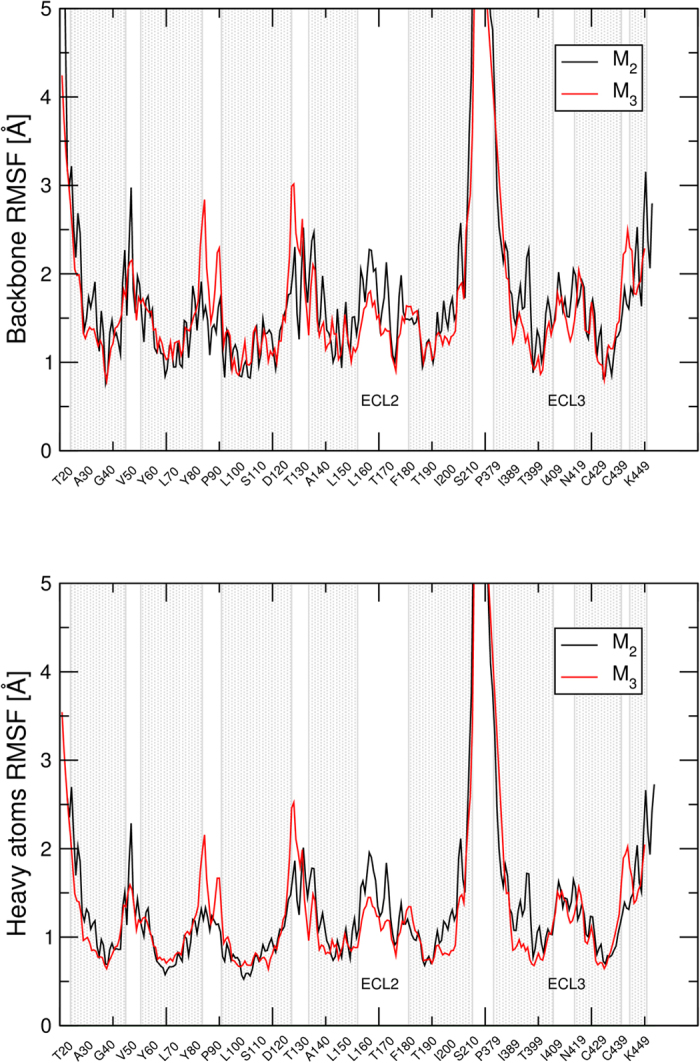
The Root Mean Square Fluctuation (RMSF) of the M_2_ and M_3_ receptor. The RMSF of backbone atoms (top) and heavy atoms (bottom) of individual amino acids of the M_2_ (black) and M_3_ (red) receptors during simulation of molecular dynamics are plotted. Grey regions represent α-helices. The second and third extracellular loops are labelled ECL2 and ECL3, respectively.

**Figure 6:**
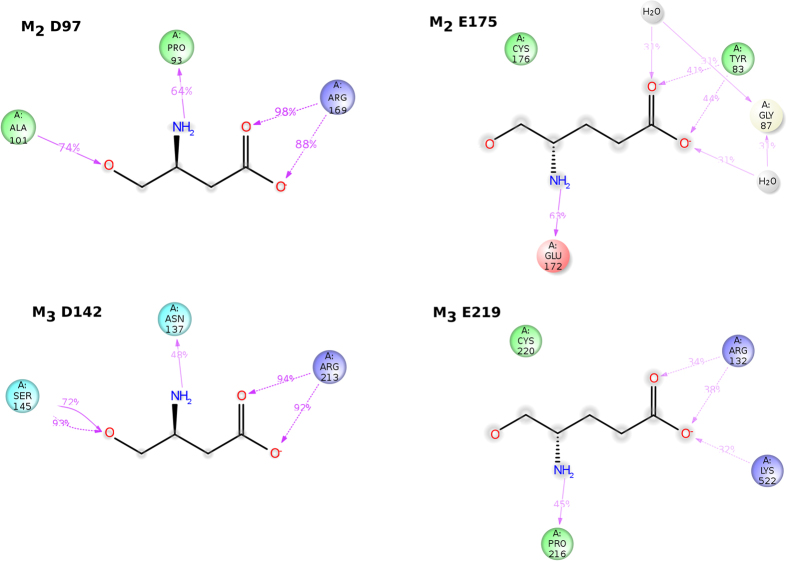
Intramolecular interactions of D97 and E175 of the M_2_ receptor and D142 and E220 of the M_3_ receptor. A schematic of detailed atom interactions of D97 (top left) and E175 (top right) of the M_2_ receptor and D147 (bottom left) and E220 (bottom right) of the M_3_ receptor with other protein residues. Interactions that occur more often than 30% of the simulation time are shown. Red – charged negative, blue – charged positive, cyan – polar, green – hydrophobic, dotted arrow – H-bond to side chain, full arrow – H-bond to backbone.

Free molecular dynamics (MD) has shown that the ECL2 and ECL3 of the M_2_ receptor are more flexible (have higher RMSF) than the ones of the M_3_ receptor (Fig. 5). This higher flexibility of the M_2_ receptor extends to the TM 5 and TM 6 α-helices. Higher flexibility corresponds to faster kinetics of [^3^H]NMS and [^3^H]QNB and to their lower affinity for the orthosteric binding site of the M_2_ than the M_3_ receptor. Analysis of intramolecular interactions of the M_2_ and M_3_ receptor over free MD trajectory highlighted two conserved amino acids that markedly contribute to the stability of the extracellular domain of the M_3_ receptor. One is D142 (D^3.26^) at the extracellular edge of TM 3 that interacts with R213 (R^4.92^) in the ECL2 and the second is E219 (E^4.99^) in the middle of the ECL2 that interacts with R132 (R^2.69^) in the ECL1 and K522 (K^7.32^) in the ECL3 (Fig. 6). Although the hydrogen bond of E219 (E^4.99^) to K522 (K^7.32^) is not present in the crystal structure, it is readily formed during MD. Interactions between E219 (E^4.99^) and K522 (K^7.32^) (hydrogen bonds as well as ionic interactions) results in stabilization of the extracellular domain of the M_3_ receptor and prevents interaction of ligands with these two amino acids. This probably explains the low affinity of the allosteric site of the M_3_ receptor for NMS.

**Figure 7:**
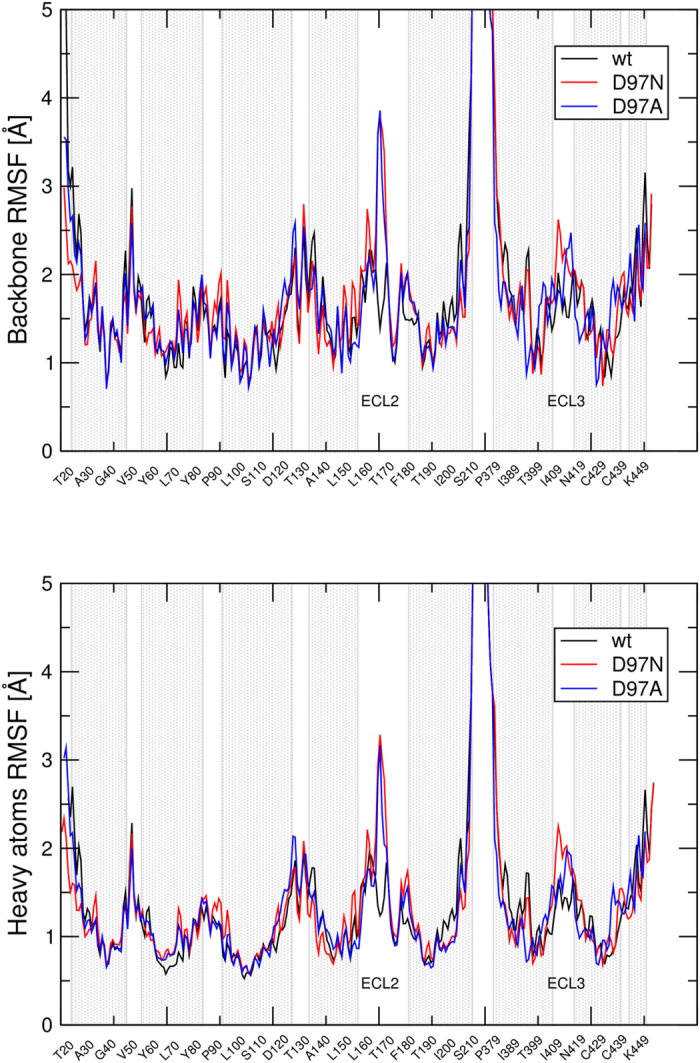
The Root Mean Square Fluctuation (RMSF) of wild type and mutated M_2_ receptor. The RMSF of backbone atoms (top) and heavy atoms (bottom) of individual amino acids of the wild type (black), D97N (red) and D97A (blue) M_2_ receptors during simulation of molecular dynamics are plotted. Grey regions denote α-helices. The second and third extracellular loops are labelled ECL2 and ECL3, respectively.

**Figure 8:**
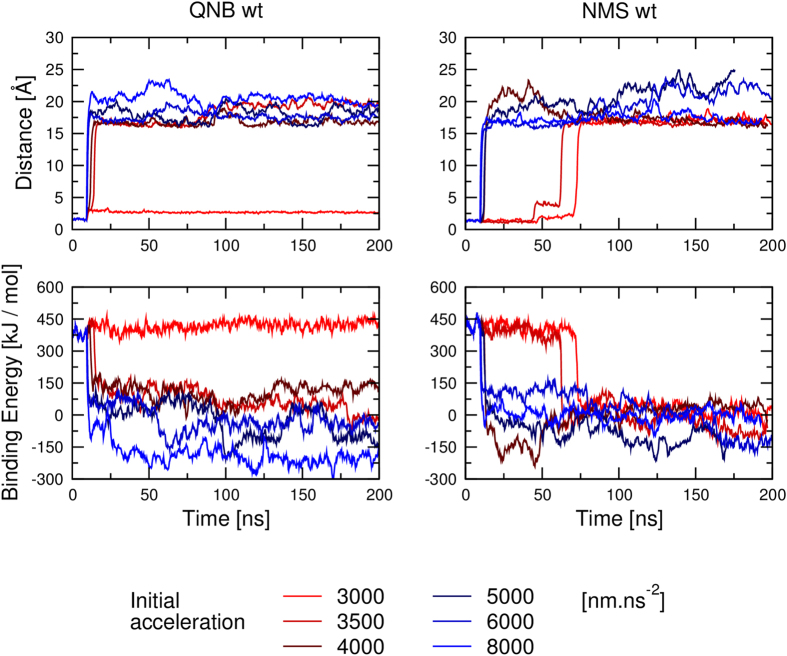
Effect of acceleration magnitude on molecular dynamics of steered dissociation. Acceleration inversely proportional to the distance of ligand centre from the bottom of the binding pocket (see Methods) was applied to QNB (left) or NMS (right) during steered molecular dynamics. Initial acceleration is indicated in the legend. Median distance of ligand centre from the bottom of binding pocket in Å (top) and ligand binding energies in kJ/mol (bottom) are plotted against simulation time.

Similar to the M_3_ receptor, D^3.26^ (D97) of the M_2_ receptor interacts with R^4.92^ (R169) in the ECL2 and stabilizes the extracellular domain (Fig. 6). In the tandem two-site model^23^ this D^3.26^ was suggested to covalently bind benzilylcholine mustard (BCM) that stops dissociation of orthosteric ligands. However, structural analysis of free MD trajectories has shown that D^3.26^ is not accessible for ligand binding. The conclusion of the original study that BCM binds to D^3.26^ was based on finding that mutation of D^3.26^ to asparagine restores dissociation of orthosteric ligands after BCM treatment. As expected, simulation of free MD of D^3.26^ (D97) mutated M_2_ receptor has shown an increase in the flexibility of the extracellular domain, especially of the ECL2 (Fig. 7). Thus explanation of dissociation of orthosteric ligands after BCM treatment from D^3.26^ mutants but not from the wild type receptor is that BCM covalently binds to some acidic residue in the ECL2, where it hinders dissociation of orthosteric ligands from the wild type receptor. However, the ECL2 is so flexible at D^3.26^ mutants that bound BCM is no longer an obstacle for orthosteric ligands when they dissociate from the receptor.

Additional amino acids in the extracellular domain potentially affecting NMS and QNB binding to the allosteric site were identified in simulation of MD of steered NMS and QNB dissociation. According to MD of steered dissociation, NMS makes hydrogen bonds primarily with E172, E175 (ECL2) and N419 (ECL3) and π-π interactions with Y177 (Figs 11 and 12) at the vestibule of the binding pocket. Importantly, mutation D97N reduces the frequency of these interactions (Fig. 11). This observation indicates that these interactions are conditioned by D97 and suggests its effects on structure-dynamics. MD of the binding of the second molecule of ligand in the vestibule to the binding pocket shows that the binding to wt receptors is stable (Fig. 13, last 10 ns) but the binding to D97N mutant is much weaker (Fig. 14, last 10 ns). In accordance with molecular modelling, mutation D97N as well as D97A accelerated [^3^H]NMS dissociation and decreased affinity of NMS for the allosteric binding site (Fig. 16, Table 3). Further, mutations of E172A, E175A and N419A accelerated [^3^H][NMS dissociation and decreased affinity of NMS for the allosteric as well as the orthosteric binding site. This is in accordance with the tandem two-site model^23^ that implies that apparent equilibrium dissociation constant (K_D_) of the orthosteric ligand is a product of equilibrium dissociation constant for the allosteric site (K_A_) and the ratio of binding to allosteric and orthosteric binding sites under equilibrium.

Glutamate in the middle of the ECL2 (E^4.99^) is present at the M_2_, M_3_ and M_5_ receptors. While at M_3_ receptor interaction of E^4.99^ with K^7.32^ (K522) (Fig. 6) prevents its interaction with NMS, the E^4.99^ is free to interact with NMS and may contribute to the high affinity of the allosteric site for NMS at the M_2_ and M_5_ subtypes. At the corresponding position in the middle of ECL2 (4.99), M_1_ and M_4_ receptors possess glutamine that is long enough to interact with E^7.32^ (M_1_) or D^7.32^ (M_4_) and may have similar effects on NMS binding as E^4.99^ at the M_3_ receptor. That is in accordance with experimental data (Table 1) demonstrating relatively low affinity of the allosteric site for NMS and slower kinetics than at the M_2_ receptor. In the position corresponding to E172 (E^4.96^) of the M_2_ receptor, other subtypes have either leucine (M_1_) or proline. E172 of the M_2_ receptor may thus contribute to the relatively high affinity of the allosteric site for NMS. Data show that N419 (N^7.32^) of the M_2_ receptor largely contributes to NMS binding to the allosteric site (Fig. 16, Table 3). However, the M_5_ receptor that has the highest affinity of allosteric site for NMS and extremely slow kinetics (Table 1) has non-polar valine at position 7.32. High affinity of the allosteric site of the M_5_ receptor for NMS is thus based on a different set of interactions than at the M_2_ receptor. In contrast to the M_3_ receptor, slow kinetics of the M_5_ receptor is not the result of stabilizing interaction between ECL3 and ECL2.

**Figure 9:**
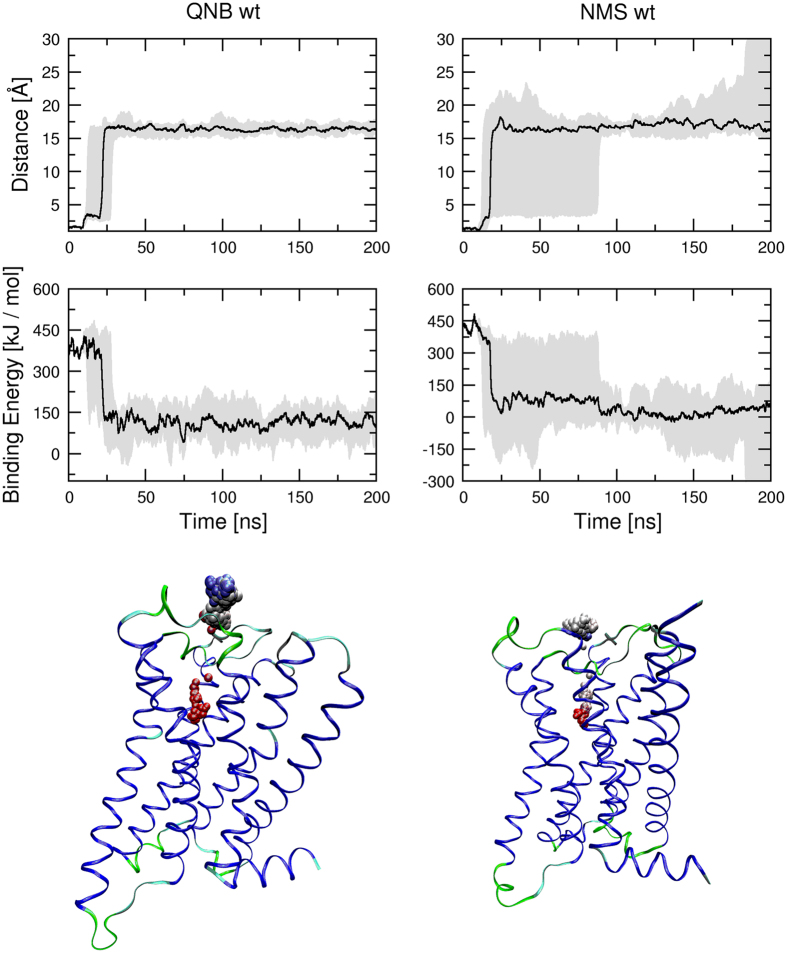
Effect of acceleration direction on molecular dynamics of steered dissociation. Acceleration inversely proportional to the distance of ligand centre from the bottom of binding pocket of the wild type M_2_ receptor (see Methods) was applied to QNB (left) or NMS (right) during MD of steered dissociation. Initial acceleration applied to ligand was 4000 nm.ns^−2^. Minimum to maximum (grey area) and median (black line) distance of ligand centre from the bottom of binding pocket in Å (top) and ligand binding energies in kJ/mol (middle) are plotted against simulation time. Bottom: Sample trajectory of steered dissociation of QNB (left) and NMS (right). Spheres representing the centre of ligand at individual time-frames are coloured according to time-frame in red to blue gradient. Videos of sample trajectories of QNB and NMS are available as Supplementary Video 1 and Supplementary Video 2, respectively.

**Figure 10:**
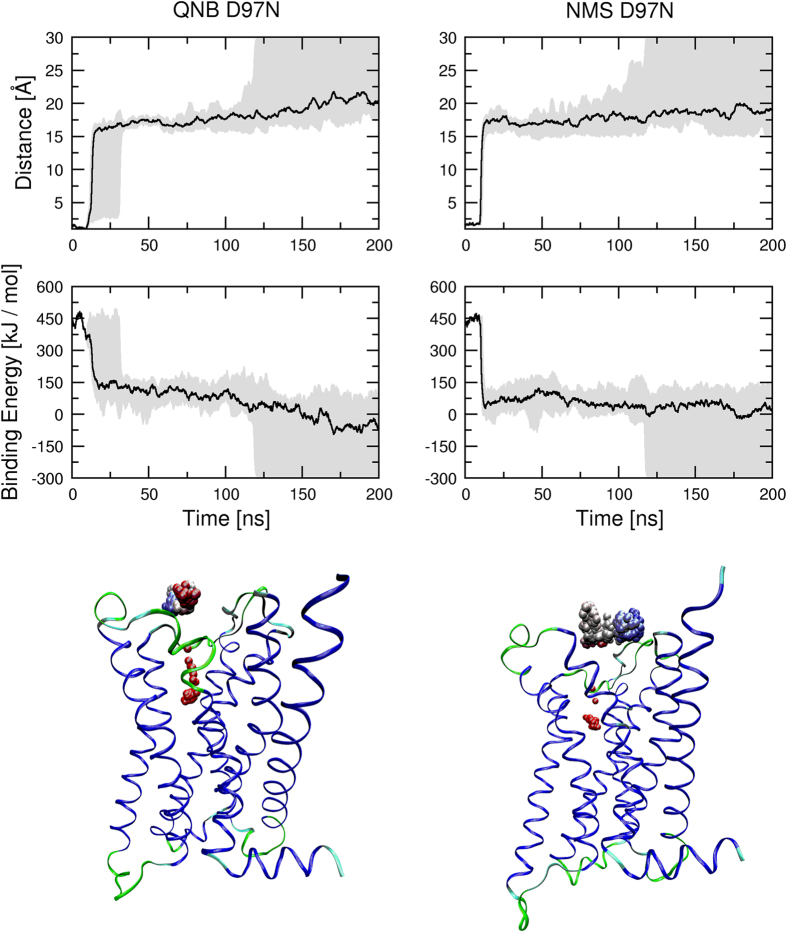
Molecular dynamics of steered dissociation from the D97N mutant. Acceleration inversely proportional to the distance of ligand centre from the bottom of binding pocket of D97N mutant M_2_ receptor (see Methods) was applied to QNB (left) or NMS (right) during MD of steered dissociation. Initial acceleration applied to ligand was 4000 nm.ns^−2^. Minimum to maximum (grey area) and median (black line) distance of ligand centre from the bottom of binding pocket in Å (top) and ligand binding energies in kJ/mol (middle) are plotted against simulation time. Bottom: Sample trajectory of steered dissociation of QNB (left) and NMS (right). Spheres representing the centre of ligand at individual time-frames are coloured according to time-frame in red to blue gradient. Videos of sample trajectories of QNB and NMS are available as Supplementary Video 3 and Supplementary Video 4, respectively.

**Figure 11:**
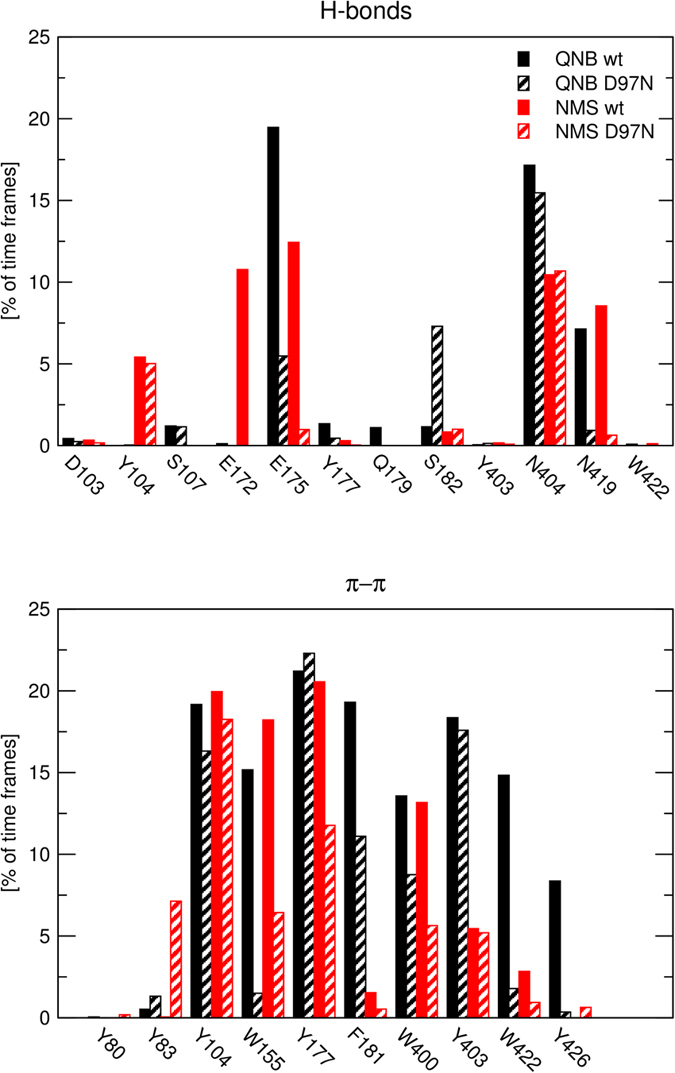
Ligand-receptor interactions during molecular dynamics of steered dissociation. Interactions of QNB (black) and NMS (red) via hydrogen bonds (top) and π-π stacking (bottom) with wild type (solid bars) or D97N mutated (hatched bars) M_2_ receptor normalized over the course of the trajectory are shown for individual amino acids. Values over 100 are possible as ligand may make multiple contacts with the receptor.

**Figure 12:**
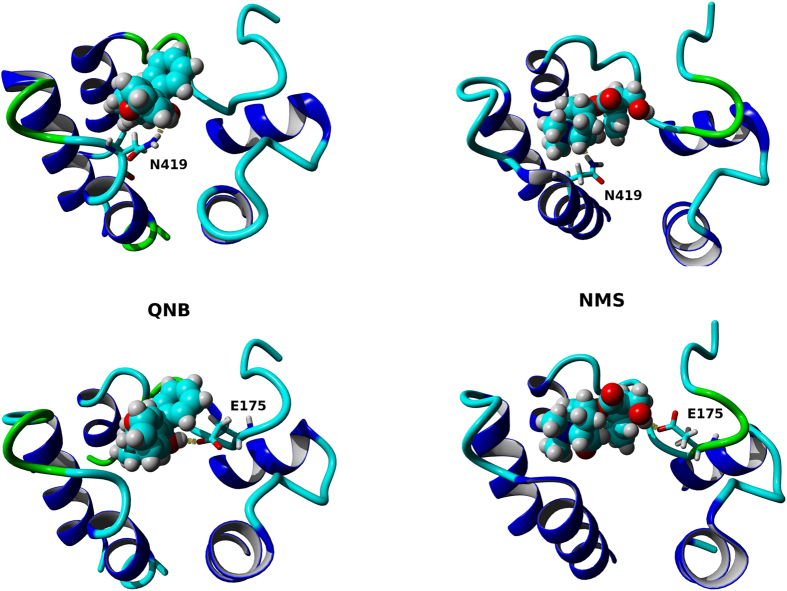
Key interactions at vestibule of the binding pocket of the M_2_ receptor. Typical placements of QNB (left) and NMS (right) interacting with key amino acids N419 (top) and E175 (bottom) at the vestibule of binding pocket of the M_2_ receptor are shown as being viewed from the extracellular side. TM 6, ECL3 and TM 7 are on the left-hand side, ECL2 is at top, TM 2, ECL1 and TM 3 are on the right-hand side. Colors: Atoms: cyan – carbon, red – oxygen, blue – nitrogen, white – hydrogen; Structures: blue – α-helix, cyan – coil; green – turn; Interactions: yellow – hydrogen bonds. QNB makes hydrogen bonds to N419 and E175, respectively, by its hydroxy group. NMS makes hydrogen bonds with N419 by its oxygen in azatricycle group, and with E175 by its hydroxy group.

**Figure 13:**
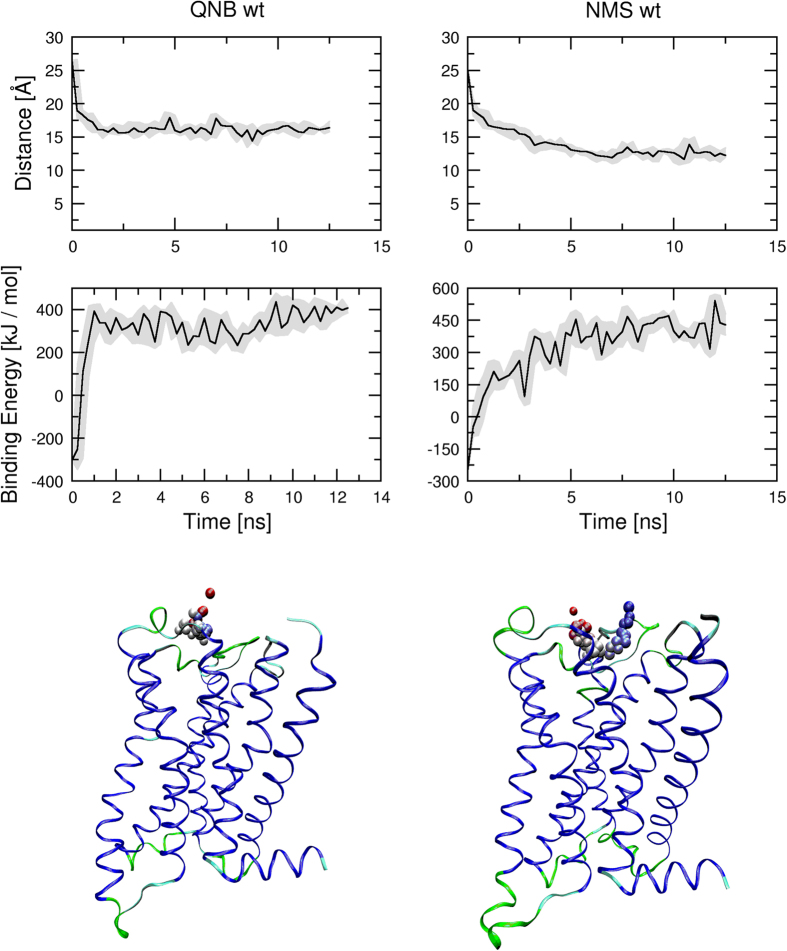
Molecular dynamics of steered association with the wild type receptor. The second molecule of QNB (left) or NMS (right) was placed to the vestibule of the binding pocket by simulation of 2.5 ns MD of steered association. Acceleration proportional to the distance of ligand centre from the bottom of binding pocket (see Methods) was applied to the second molecule of the ligand. Initial acceleration applied to ligand was 500 nm.ns^−2^. Then 10 ns of free MD followed. Minimum to maximum (grey area) and median (black line) distance of ligand centre from the bottom of binding pocket in Å (top) and ligand binding energies in kJ/mol (middle) are plotted against simulation time. Bottom: Sample trajectory of steered association of QNB (left) and NMS (right). Spheres representing the centre of ligand at individual time-frames are coloured according to time-frame in red to blue gradient. Videos of sample trajectories of QNB and NMS are available as Supplementary Video 5 and Supplementary Video 6, respectively.

**Figure 14:**
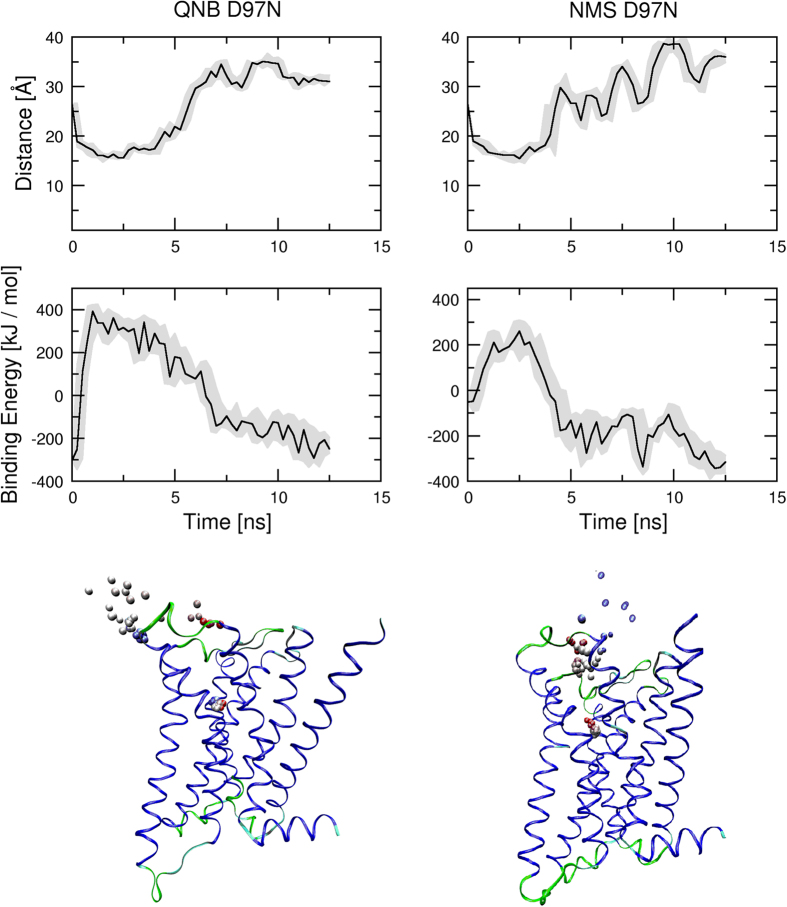
Molecular dynamics of steered association with the D97N mutant. The second molecule of QNB (left) or NMS (right) was placed to the vestibule of the binding pocket by simulation of 2.5 ns MD of steered association. Acceleration proportional to the distance of ligand centre from the bottom of binding pocket (see Methods) was applied to the second molecule of the ligand. Initial acceleration applied to ligand was 500 nm.ns^−2^. Then 10 ns of free MD followed. Minimum to maximum (grey area) and median (black line) distance of ligand centre from the bottom of binding pocket in Å (top) and ligand binding energies in kJ/mol (middle) are plotted against simulation time. Bottom: Sample trajectory of steered association of QNB (left) and NMS (right). Spheres representing the centre of ligand at individual time-frames are coloured according to time-frame in red to blue gradient. Videos of sample trajectories of QNB and NMS are available as Supplementary Video 7 and Supplementary Video 8, respectively.

**Figure 15:**
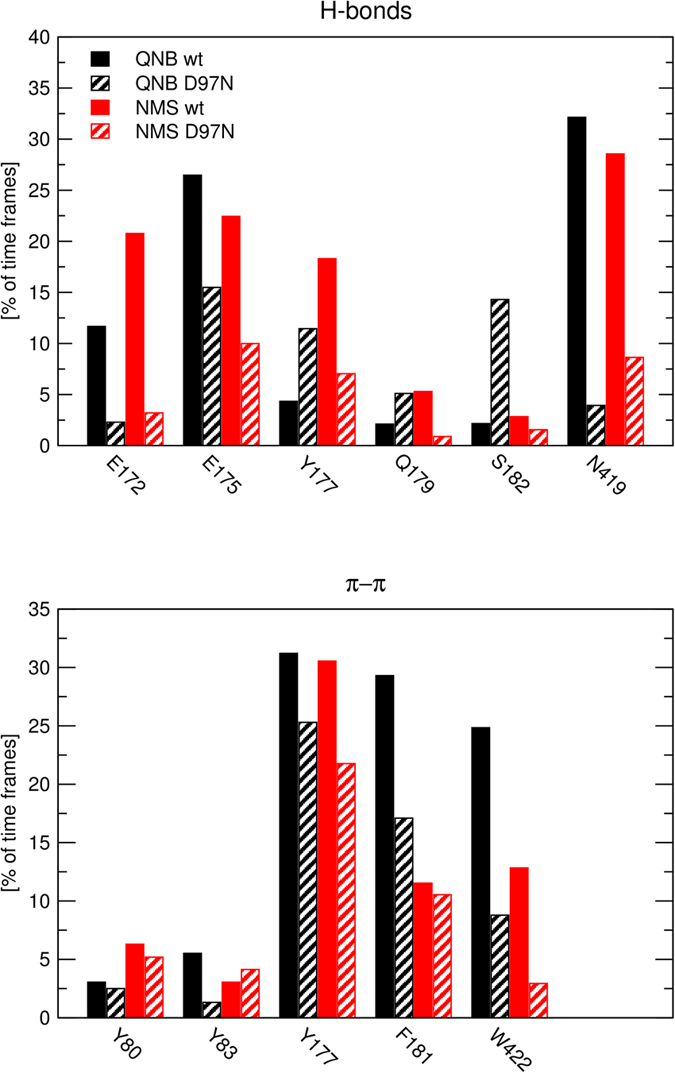
Ligand-receptor interactions during molecular dynamics of steered association. Interactions of QNB (black) and NMS (red) via hydrogen bonds (top) and π-π stacking (bottom) with wild type (solid bars) or D97N mutated (hatched bars) M_2_ receptor normalized over the course of the trajectory are shown for individual amino acids. Values over 100 are possible as ligand may make multiple contacts with the receptor.

**Figure 16:**
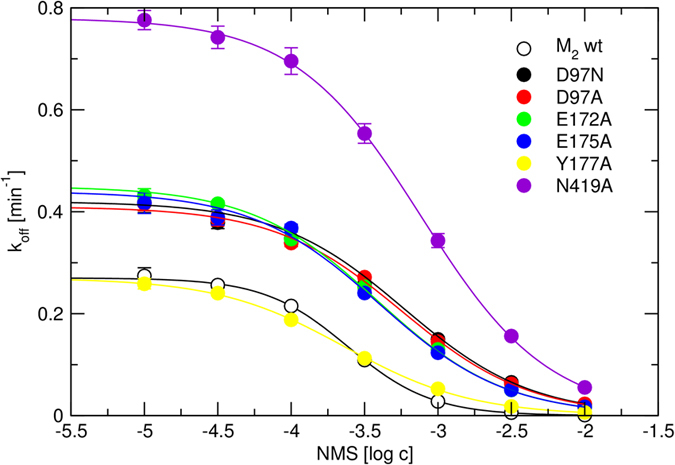
Dissociation rate constants of [^3^H]NMS from mutated M_2_ receptors. Dissociation rate constants (k_off_) of [^3^H]NMS from mutated M_2_ receptors calculated from time-courses according Eq. 2 or Eq. 3 are plotted against the concentration of cold NMS used to initiate [^3^H]NMS dissociation. Eq. 4 or Eq. 5 was fitted to data to estimate K_A_ (Table 3). Data are means ± S.E.M. from 4 independent experiments performed in duplicates.

The extracellular domain of muscarinic receptors has been shown to be essential in binding of many allosteric modulators^7^,^8^,^9^,^10^,^11^,^12^,^13^,^14^, ectopic ligands^15^ and bitopic ligands^16^,^17^,^18^. For example, glutamates E172 and E175 with which NMS interacts are part of the EDGE motif that has been shown to be essential for binding of the prototypic allosteric modulator gallamine^7^. In the crystal structure of the M_2_ receptor^13^ the allosteric modulator LY2119620 does not form a hydrogen bond to E172, E175 nor N419 but, similarly to NMS, makes π-π interactions to Y177 and Y426. Two centres for binding of positive nitrogen were identified in computer modelling studies of allosteric ligand binding to M_2_ receptors^34^. The first centre consists of Y177, N410, N419 and W422 and the second consists of Y80, Y83, T84 and T423. Further, this study shows that dimethyl-W84, gallamine, alcuronium and strychnine bind to the fist centre. The presented data indicate that NMS also binds to the first centre. From the study of Redka *et al*.^24^ it could be concluded that this very site also binds the allosteric modulator obidoxime and the orthosteric agonist oxotremorine-M. The site is located next to the opening of a tyrosine lid on top of the orthosteric site and our molecular modelling studies (Fig. 9) suggest that all pathways to and from the orthosteric binding site go through this extracellular site that is thus common for all orthosteric and many allosteric ligands. This indicates that binding of orthosteric ligands to the allosteric site is mutually exclusive with binding of some allosteric, ectopic and bitopic ligands. Interpretation of binding data from kinetic experiments needs to account for this phenomenon. Mutations in the extracellular domain may affect binding kinetics of radiolabeled orthosteric tracers, which complicates interpretation of effects of mutagenesis on binding of an unlabelled ligand. Discrimination between effects of mutation on tracer binding and on tested ligand binding is essential for elucidation of molecular mechanisms of binding of tested ligand.

Although the apparent affinity of orthosteric ligands for the first extracellular centre mentioned above is very low, it is important to note that any orthosteric ligand has to “travel” through this site. Consequently, ligand interaction with this site affects kinetics of orthosteric agonists and thus their residence time at the receptor that then impacts receptor activation^35^. Further, allosteric activators of muscarinic receptors have been described. Thus, an orthosteric agonist that activates the receptor also from the extracellular site may exist. Taken together binding to the extracellular site may affect functional properties of orthosteric agonists.

In summary, orthosteric ligands also interact with the allosteric site located in the vestibule to the binding pocket of muscarinic receptors between ECL2 and ECL3 on their way to and from the orthosteric binding site. This site overlaps with the binding site of some allosteric, ectopic and bitopic ligands. Namely, NMS interacts with E172 and E175 and N419 at the M_2_ muscarinic receptor. Conserved D97 (D^3.26^) stabilizes the conformation of ECL2. Proper conformation of ECL2 is a prerequisite for optimal binding of NMS to the allosteric binding site. Transient binding of orthosteric ligands to this extracellular site may have pharmacologically relevant consequences.

## Methods

### Chemicals

Tritiated quinuclidinyl benzilate [benzilic-4.4-^3^H(N)] ([^3^H]QNB) and N-[methyl-^3^H]methylscopolamine ([^3^H]NMS) were from ARC (St. Louis, MO), non-labelled quinuclidinyl benzilate (QNB) and N-methylscopolamine (NMS) were from Sigma (Prague, Czech Republic).

### Residues naming and numbering

Single amino acid mutants are named by a single letter code and position number of the mutated amino acid followed by a single letter code of the amino acid mutated to. For the sake of brevity names of mutants of M_3_ receptor in the third extracellular loop (ECL3) consist of the receptor subtype name followed by a lists of mutated amino acids without position numbers. Amino acids in the ECL2 of M_3_ receptor were always mutated to the corresponding residues in the M_2_ receptor subtype. For example, M_3_ SK- > PN means that serine 519 and lysine 523 of the M_3_ receptor were mutated to the corresponding proline and asparagine in the M_2_ sequence and M_3_ DSKFN- > APNVT means that five amino acids in the ECL3 of the M_3_ receptor were mutated to their corresponding residues in the M_2_ sequence (See Supplementary Information, Figure SI1). Ballesteros Weinstein numbering was used^30^ when comparing residues of different receptors. In addition position numbering from conserved cysteine in ECL2 was used for position numbering of residues in ECL2 (that varies in the length). This conserved cysteine was assigned number 4.100.

### Mutagenesis and Expression

The mammalian expression vector pcDNA3.1 (Invitrogen, Carlsbad, CA, USA) containing the coding sequence of the human variants of the M_2_ and M_3_ subtype of muscarinic acetylcholine receptors was obtained from Missouri S&T cDNA Resource Center (Rolla, MO, USA). Construction of mutants was previously described in Krejčí and Tuček^9^ and Jakubik *et al*.^17^. Additional mutants were generated using QuikChange II Site-Directed Mutagenesis Kit (Agilent Technologies Company, Santa Clara, CA, USA).

CHO cells were transfected with the use of Lipofectamine 2000 (Life Technologies, Praha, Czech Republic) according to manufacturer’s guidelines. They were grown in 10-cm Petri dishes in Dulbecco’s modified Eagel’s medium (Sigma) with 10% fetal calf serum. On day one 2 × 10^6^ cells were seeded per dish. On day three after washing with phosphate-buffered saline, the transfection mixture containing 1 μg of plasmid DNA and 5 μl of Lipofectamine in 1 ml of Opti-MEM (Life Technologies, Praha, Czech Republic) was applied for 6 h. Transfection medium was then removed and fresh medium supplemented with 10% fetal calf serum was applied. Cells were harvested 72 h after transfection.

### Radioligand Binding Experiments

Radioligand binding experiments were performed on membranes from CHO cells. Membranes were obtained by dilution of freshly harvested CHO cells in homogenization buffer composed of 100 mM NaCl, 10 mM EDTA and 10 mM Na-HEPES (pH = 7.4) to a final concentration of 10^7^ cells per ml. Cell suspension was homogenized using Ultra-Turrax homogenizer by two 30 second strokes. Homogenate was centrifuged 5 min at 1,000 × g. Resulting supernatant was centrifuged for 30 min at 30,000 × g and supernatant was discarded. Pellet was resuspended in 10 times of the original volume (before centrifugation) of incubation buffer composed of 100 mM NaCl, 10 mM MgCl_2_ and 10 mM Na-HEPES (pH = 7.4), left at 4 °C for 30 min and then the latter centrifugation step was repeated. Membranes were kept frozen at −20 °C for a maximum of one month. Radioligand binding experiments were carried out in 96-well plates and incubation volume was 0.8 ml in case of saturation binding and 0.2 ml in case of kinetic experiments. Incubations performed at 25 °C were terminated by filtration through Whatman GF/C glass fiber filters in a Brandel filtration apparatus (Sensat, Herts, UK). Non-specific binding of [^3^H]QNB and [^3^H]NMS was determined in the presence of 1 μM atropine.

The affinity of wild-type and mutated muscarinic receptors for [^3^H]QNB and [^3^H]NMS was measured in saturation binding experiments with [^3^H]QNB at concentrations ranging from 16 pM to 500 pM or with [^3^H]NMS at concentrations ranging from 32 pM to 1 nM. The incubation lasted 12 h to achieve full equilibrium. In dissociation experiments, membranes were preincubated for 12 h with 500 pM [^3^H]QNB or 1 nM [^3^H]NMS. Dissociation was induced by adding of QNB or NMS to the desired final concentrations.

### Data treatment

Data were processed and analysed with open source software OpenOffice 3.4 (OpenOffice Foundation, www.openoffice.org) and Grace 5.1.12 (Grace Development Team, plasma-gate.weizmann.ac.il/Grace) on Scientific Linux (www.scientificlinux.org).

The equations for non-linear regression analysis were as follows:

For saturation binding experiments:

After subtraction of non-specific binding Eq. 1 was fitted to the data.


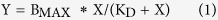


where Y is [^3^H]NMS binding at concentration X of free [^3^H]NMS, K_D_ is the equilibrium dissociation constant, and B_MAX_ is the number of binding sites.

For dissociation experiments:

After subtraction of non-specific binding and normalization to binding in the beginning of dissociation Eq. 2 and Eq. 3 were fitted to the data.


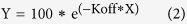



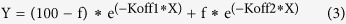


where Y is [^3^H]NMS binding at time X, and k_off_ is the observed dissociation rate constant, and f is percentage of sites displaying dissociation rate of k_off2_.

Apparent equilibrium dissociation constant K_A_ for NMS or QNB binding to the secondary binding site based on dissociation experiments was obtained according Lazareno and Birdsall^33^ and Eq. 4 or Eq. 5 was fitted to the data:


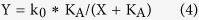



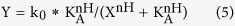


where Y is observed rate of dissociation k_off_ at concentration X of cold QNB or NMS and k_0_ is dissociation rate induced by 1 μM QNB or NMS and nH is a slope factor.

Statistical data analysis was performed using the statistical package R (http://www.r-project.org/).

### Molecular modeling

#### Preparation of structures and systems

Crystal structures of the M_2_ (PDB code 3UON) and M_3_ (PDB code 4DAJ) receptors^5^,^6^ were downloaded from RCSB Protein Data Bank (www.rcsb.org), and pre-processed with Schrödingers’ Protein Preparation Wizard to remove non-receptor parts, fill missing side chains and energy minimize structures in OPLSA 2005 force field. Single amino acid mutations were constructed by FoldX^36^. Structure of N-methylscopolamine (CID 656633) was downloaded from PubChem database (pubchem.ncbi.nlm.nih.gov) and processed with Shrödingers’ LigPrep. NMS was docked to the M_2_ and M_3_ receptors and QNB was cross-docked to the M_3_ receptor using Glide followed by Prime-MM/GBSA re-scoring as described earlier^37^.

#### Simulation of molecular dynamics

A system consisting of receptor with bound QNB, POPE (1-palmitoyl-2-oleoyl phosphatidylethanolamine) membrane, water and 0.15 M NaCl was built with Desmond System Builder. Molecular dynamics of full membrane systems was simulated using Desmond^38^. Aspartates D2.50 and D3.32 were protonated. First, systems were relaxed to 300 K using standard Desmond protocol for membrane systems (that prevents water and ions from entering the membrane) and subsequently 120 ns of free (without restrains) molecular dynamics (MD) (ensemble class NVE, Coulombic short range method - cutoff with radius 9 Å, long range method – smooth particle mesh Evald) was simulated using Desmond-GPU.

#### Steered molecular dynamics

Alternatively, MD of steered ligand dissociation from the orthosteric binding site or steered ligand association with the secondary site was simulated using YASARA^39^. A system consisting of the M_2_ receptor with bound QNB or NMS, POPE membrane, water and 0.15 M NaCl was built with script provided by YASARA with default setting. Aspartates D2.50 and D3.32 were protonized. Then system was equilibrated and 10 ns free MD at 300 K was run by YASARA script for membrane proteins (that prevents water and ions entering the membrane). After that either MD of steered dissociation was run or second ligand was placed on the extracellular side from the receptor. During steered dissociation (200 ns or till complete ligand dissociation) acceleration inversely proportional to the distance between centre of ligand and centre of bottom of binding pocket in the direction from the bottom of binding pocket was applied to the ligand every 100^th^ step of MD. The bottom of binding pocket was defined as S107, A194 and W400. Alternatively, to study effects of acceleration direction, the bottom of the binding pocket was defined as any combination of two aforementioned amino acids. The initial value of acceleration (before division by distance) ranged from 3500 to 8000 nm.ns^−2^, and resulting acceleration was capped at 4000 nm.ns^−2^. During steered association (2.5 ns) acceleration proportional to the distance (1/(30 - distance) between centre of ligand and centre of bottom of binding pocket in the direction towards the bottom of binding pocket was applied to the ligand every 100^th^ step of MD. The initial value of acceleration (before division by the value of 30.0 Å minus distance) was 500 nm.nm^−2^. The bottom of binding pocket was defined as S107, A194 and W400. Steered association was followed by 10 ns free MD.

#### Analysis of molecular dynamics

The quality of molecular dynamics simulation was assessed in Maestro by Simulation Quality Analysis tools, analysed by Simulation Event Analysis tool. Intramolecular interactions were identified using Simulation Interaction Diagram tool. The Root Mean Square Fluctuation (RMSF) for residue *i* was calculated as:


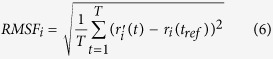


where T is the trajectory time over which the RMSF is calculated, t_ref_ is the reference time, r_i_ is the position of residue *i*; r′ is the position of atoms in residue *i* after superposition on the reference (time 0).

For every frame of steered MD the distance of the centre of ligand and the centre of binding pocket and ligand binding energy was calculated and amino acids interacting by hydrogen bonding or π-π interactions were determined using customized scripts provided by YASARA. In the YASARA binding energy function, the energy is calculated as the difference between the sum of potential and solvation energies of the separated compounds and the sum of potential and solvation energies of the complex in the YAMBER3 force field.

## Additional Information

**How to cite this article**: Jakubík, J. *et al*. Binding of N-methylscopolamine to the extracellular domain of muscarinic acetylcholine receptors. *Sci. Rep.* **7**, 40381; doi: 10.1038/srep40381 (2017).

**Publisher's note:** Springer Nature remains neutral with regard to jurisdictional claims in published maps and institutional affiliations.

## Supplementary Material

Supplementary Information

Supplementary video 1

Supplementary video 2

Supplementary video 3

Supplementary video 4

Supplementary video 5

Supplementary video 6

Supplementary video 7

Supplementary video 8

## Figures and Tables

**Table 1:** Parameters of [^3^H]NMS and [^3^H]QNB binding to individual subtypes of muscarinic receptors.

	NMS			QNB		
	pK_D_	k_off_ [min^−1^]	pK_A_	pK_D_	k_off_ [min^−1^]	k_off_ [min^−1^] @ 1 mM QNB
M_1_	9.60 ± 0.02	0.056 ± 0.007	3.52 ± 0.03	9.82 ± 0.02	1.71 × 10^−3^ ± 0.05 × 10^−3^	7.7 × 10^−4^ ± 0.2 × 10^−4^
M_2_	9.43 ± 0.02*	0.27 ± 0.02*	3.62 ± 0.03	9.80 ± 0.02	3.0 × 10^−3^ ± 0.01 × 10^−3^*	12 × 10^−4^ ± 3 × 10^−4^
M_3_	9.64 ± 0.02	0.034 ± 0.003	3.25 ± 0.02	9.87 ± 0.02	1.21 × 10^−3^ ± 0.06 × 10^−3^	6.03 × 10^−4^ ± 0.04 × 10^−4^
M_4_	9.66 ± 0.02	0.043 ± 0.004	3.31 ± 0.02	9.83 ± 0.02	1.58 × 10^−3^ ± 0.07 × 10^−3^	7.78 × 10^−4^ ± 0.04 × 10^−4^
M_5_	9.52 ± 0.02	0.0162 ± 0.0003*	3.86 ± 0.03*	9.85 ± 0.02	1.40 × 10^−3^ ± 0.05 × 10^−3^	6.26 × 10^−4^ ± 0.07 × 10^−4^

Equilibrium dissociation constants (K_D_) of NMS and QNB binding to the orthosteric site are expressed as negative logarithm. Dissociation rate constants are expressed in per minute rate and apparent affinity of NMS for the allosteric site (K_A_) is expressed as negative logarithm. It was not possible to reliably estimate apparent affinity of QNB for the allosteric site, therefore the dissociation rate of [^3^H]QNB in the presence of 1 mM QNB is shown. Data are means ± S.E.M. from 3 (equilibrium) or 4 (kinetics) independent experiments performed in triplicates (equilibrium) or duplicates (kinetics).

^*^Different (p < 0.01; ANOVA, Dunnett’s post-test) from other subtypes.

**Table 2:** Parameters of [^3^H]NMS binding to the M_3_ receptor with mutated ECL3 loop.

	pK_D_	k_off_ [min^−1^]	pK_A_
M_3_ wt	9.64 ± 0.02	0.034 ± 0.003	3.25 ± 0.02
M_3_ S- > P	9.65 ± 0.02	0.057 ± 0.003*	3.25 ± 0.03
M_3_ K- > N	9.63 ± 0.02	0.097 ± 0.004*	3.52 ± 0.02*
M_3_ KFN- > NVT	9.55 ± 0.02*	0.26 ± 0.01*	3.65 ± 0.02*
M_3_ DKFN- > ANVT	9.56 ± 0.02*	0.21 ± 0.01*	3.64 ± 0.02*
M_3_ SK- > PN	9.63 ± 0.02	0.12 ± 0.01*	3.55 ± 0.02*
M_3_ SKFN- > PNVT	9.48 ± 0.02*	0.25 ± 0.01*	3.65 ± 0.02*
M_3_ DSKFN- > APNVT	9.45 ± 0.02*	0.26 ± 0.01*	3.66 ± 0.02*
M_2_ wt	9.43 ± 0.02*	0.27 ± 0.02*	3.62 ± 0.03

Equilibrium dissociation constants (K_D_) of NMS binding to the orthosteric site are expressed as negative decadic logarithm. Dissociation rate constants are expressed in per minute rate and apparent affinity of NMS for the allosteric site (K_A_) is expressed as negative decadic logarithm. Data are means ± S.E.M. from 3 (equilibrium) or 4 (kinetics) independent experiments performed in triplicates (equilibrium) or duplicates (kinetics).

^*^Different (p < 0.05; ANOVA, Dunnett’s post-test) from M_3_ wt. Standard nomenclature of mutations: D518A, S519P, K523N, F525V, N527T.

**Table 3:** Parameters of [^3^H]NMS binding to mutated M_2_ receptor.

	pK_D_	k_off_ [min^−1^]	pK_A_
M_2_ wt	9.43 ± 0.02	0.27 ± 0.02	3.62 ± 0.03
M_2_ D97N	9.03 ± 0.02*	0.42 ± 0.04*	3.24 ± 0.02*
M_2_ D97A	9.04 ± 0.02*	0.41 ± 0.04*	3.23 ± 0.02*
M_2_ E172A	9.21 ± 0.02*	0.45 ± 0.04*	3.39 ± 0.02*
M_2_ E175A	9.19 ± 0.02*	0.44 ± 0.04*	3.40 ± 0.02*
M_2_ Y177A	9.44 ± 0.02	0.27 ± 0.03	3.63 ± 0.02
M_2_ N419A	8.95 ± 0.02*	0.78 ± 0.06*	3.11 ± 0.03*

Equilibrium dissociation constants (K_D_) of NMS binding to the orthosteric site are expressed as negative logarithm. Dissociation rate constants are expressed in per minute rate and apparent affinity of NMS for the extracellular site (K_A_) is expressed as negative logarithm. Data are means ± S.E.M. from 3 (equilibrium) or 4 (kinetics) independent experiments performed in triplicates (equilibrium) or duplicates (kinetics).

^*^Different (p < 0.05; ANOVA, Dunnett’s post-test) from M_2_ wt.
