# Supplementary material for: Binding of N-methylscopolamine to the extracellular domain of muscarinic acetylcholine receptors
Source: Sci Rep. 2017 Jan 16;7:40381. doi: 10.1038/srep40381 (PMC5238504; doi:10.1038/srep40381)
Supplement: Supplementary Information [file srep40381-s1.pdf]

Supplementary information: Binding of N-methylscopolamine to the extracellular domain of muscarinic acetylcholine receptors

Jan Jakubík, Alena Randáková, Pavel Zimčík, Esam E. El-Fakahany, and Vladimír Doležal

Figure SI1: Sequence alignments of the ECL3 of M_2 and M_3 receptors.

M_2 ^{409}N T F C **A** **P** C I P **N** T **V** W **T**^{423}

M_3 ^{514}N T F C **D** **S** C I P **K** T **F** W **N**^{527}

Figure SI2: Dissociation of [^{3}H]QNB induced by NMS

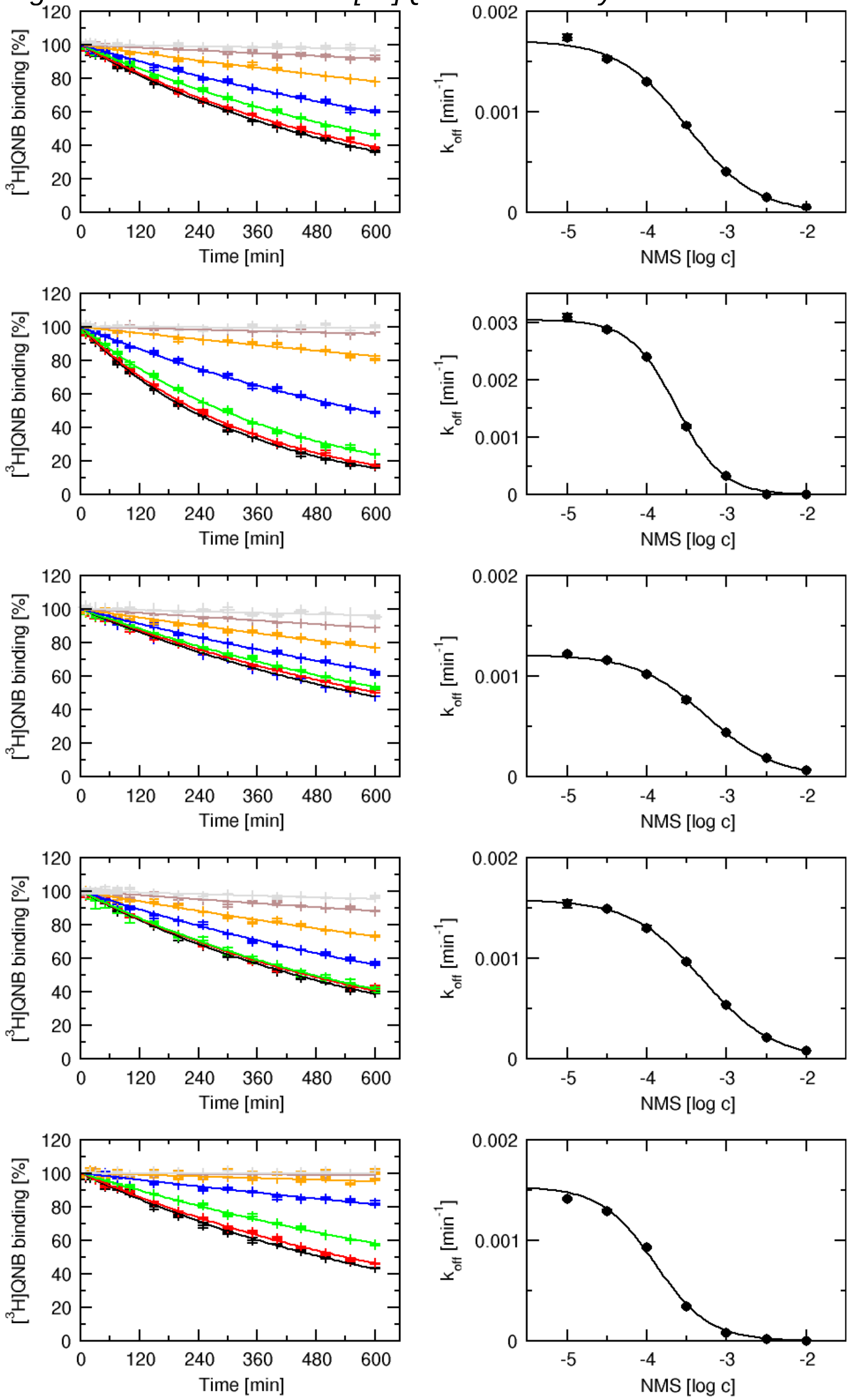

Time course of dissociation of 500 pM [^{3}H]QNB from individual receptor subtypes (left) initiated by the addition of NMS at final concentrations varying from 10 μM (black) to 10 mM (gray) was followed for the time periods indicated on the abscissa. Binding of [^{3}H]QNB (ordinate) is expressed as per cent

of binding at the beginning of dissociation. Dissociation rates (k_{off}) calculated from time-courses according to Eq. 2 are plotted against the concentration of cold QNB used to initiate [^{3}H]QNB dissociation (right). Data are means ± S.E.M. from 4 independent experiments performed in duplicates.

Table SI1 Apparent affinity of NMS measured calculated from kinetics of [^{3}H]QNB dissociation.

Apparent affinity of NMS for the allosteric site (K_A) is expressed as negative logarithm. Data are means ± S.E.M. from or 4 independent experiments performed in duplicates.

	pK_A
M_1	3.50 ± 0.02
M_2	3.62 ± 0.02
M_3	3.24 ± 0.02
M_4	3.29 ± 0.02
M_5	3.82 ± 0.02

Figure SI3: Orientation of $E^{4.99}$ and $N/K^{7.32}$ in average structures from MD of M_2 and M_3 receptors

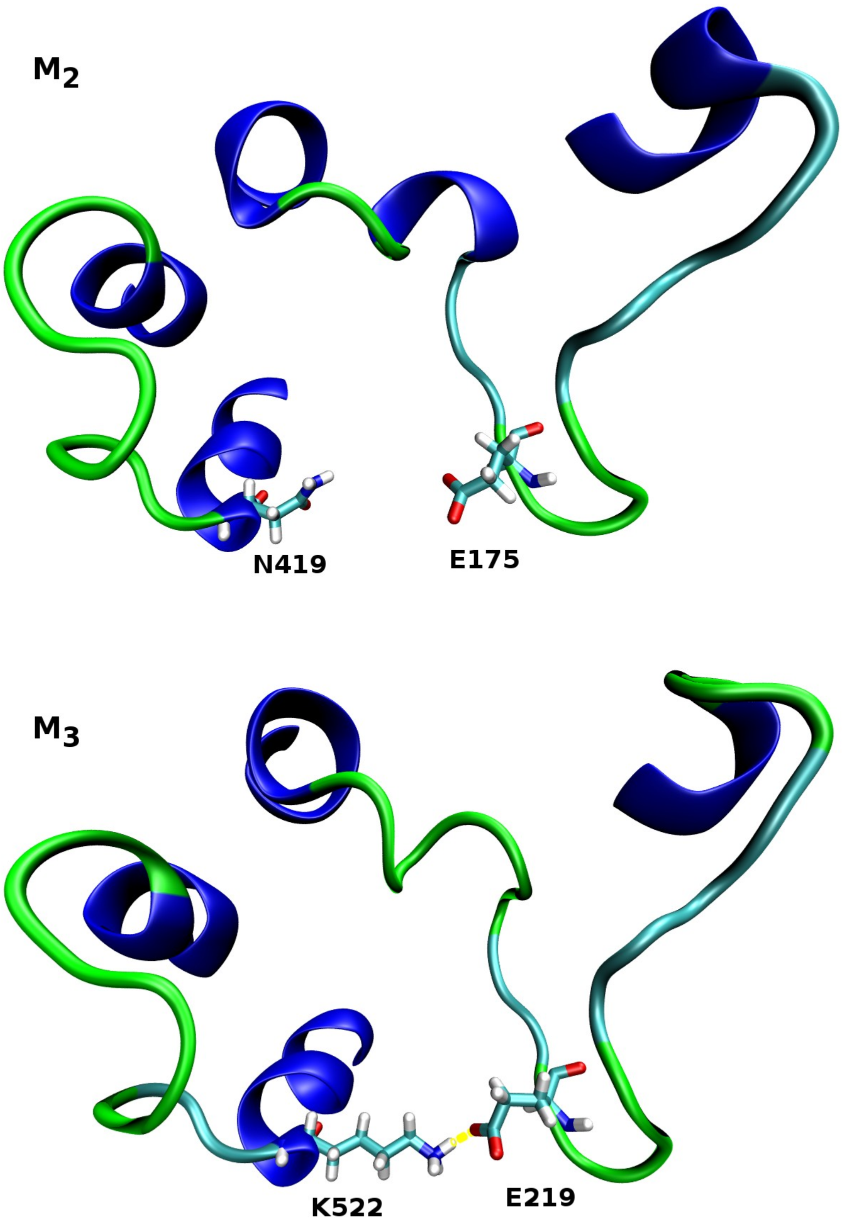

Orientation of $E^{4.99}$ and $N^{7.32}$ and $K^{7.32}$, respectively, in average structures from MD of M_2 (top) and M_3 (bottom) receptors as viewed from the extracellular side. At M_3 receptors conserved $E^{4.99}$ (E219) forms hydrogen bond with $K^{7.32}$ (K522). In contrast at M_2 receptor these two amino acids, $E^{4.99}$ (E175) and $N^{7.32}$ (N419) are free. Colours: Atoms: cyan – carbon, red – oxygen, blue – nitrogen, white – hydrogen; Structures: blue – α-helix, cyan – coil; green – turn; Interactions: yellow – hydrogen bonds.

Figure SI4: Concurrent interaction of NMS with N419 and E175

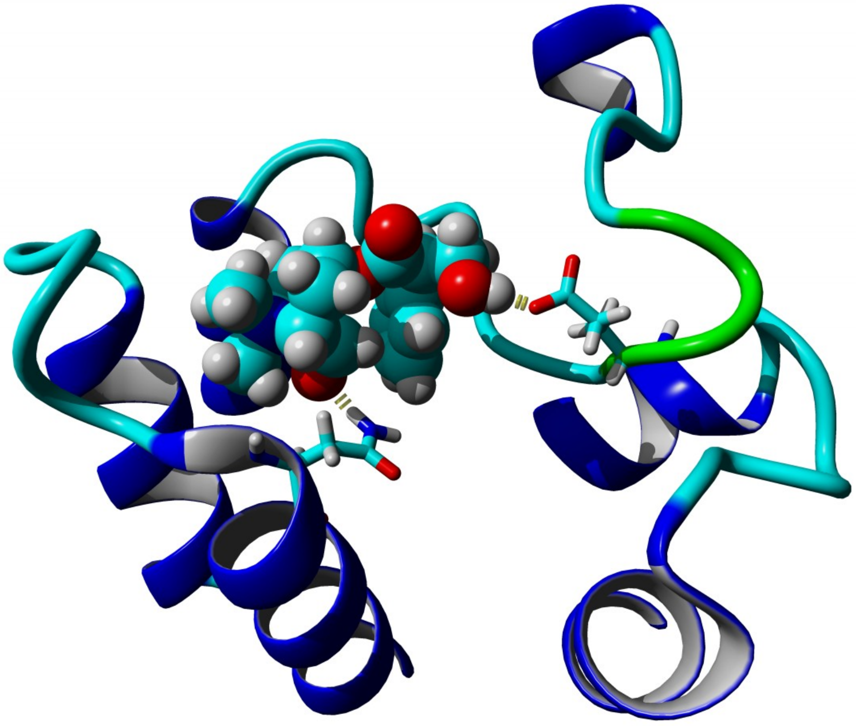

Structure of NMS interacting concurrently with key amino acids N419 and E175 at the vestibule of binding pocket of M_2 receptor is show as viewed from extracellular side. TM 6, ECL3 and TM 7 are on the left-hand side. Colours: Atoms: cyan – carbon, red – oxygen, blue – nitrogen, white – hydrogen; Structures: blue – α-helix, cyan – coil; green – turn; Interactions: yellow – hydrogen bonds.

Figure SI4: Less frequent interactions of QNB at the vestibule to binding pocket

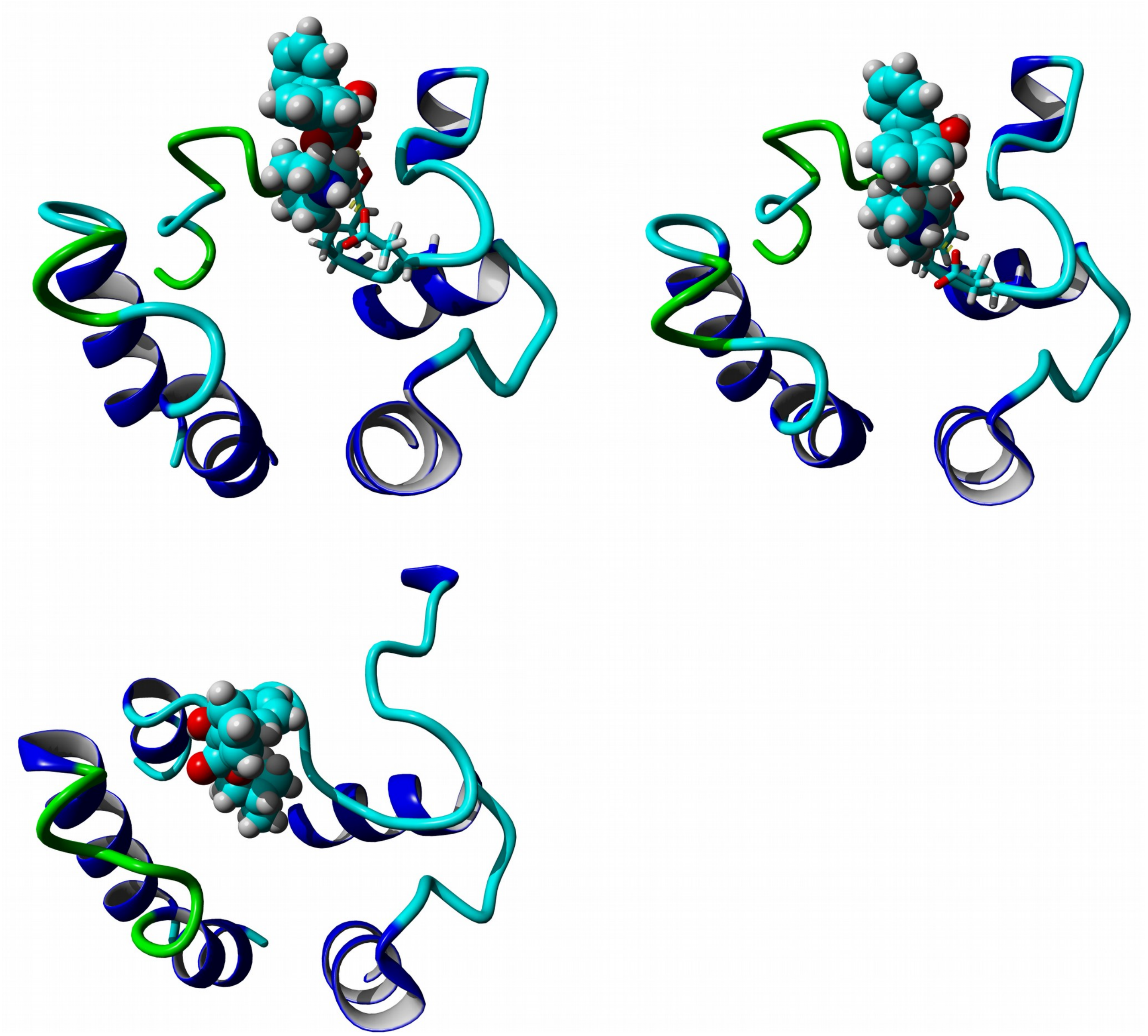

Structure of QNB concurrently interacting with E172, E175 and Q179 (top left), E175, Y177 and Q179 (top right) or interacting with S182 (bottom) at the vestibule of binding pocket of M_2 receptor is show as viewed from extracellular side. TM 6, ECL3 and TM 7 are on the left-hand side. Colours: Atoms: cyan – carbon, red – oxygen, blue – nitrogen, white – hydrogen; Structures: blue – α-helix, cyan – coil; green – turn; Interactions: yellow – hydrogen bonds.

Figure SI5: Less frequent interactions of NMS at the vestibule to binding pocket

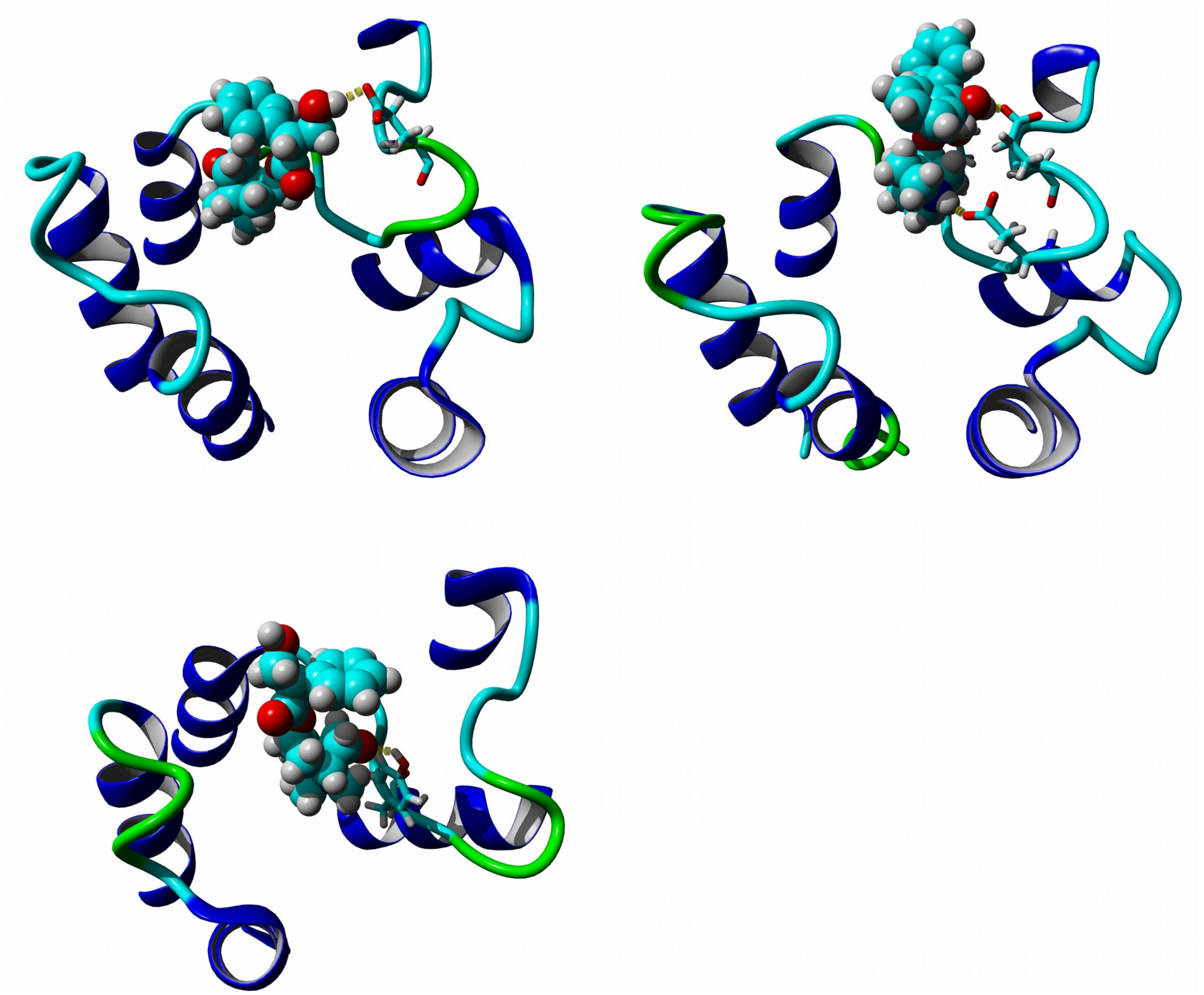

Structure of NMS interacting with E172 (top left) and Y177 (bottom) or interacting concurrently with E172 and Q179 (top right) at the vestibule of binding pocket of the M_2 receptor is show as viewed from extracellular side. TM 6, ECL3 and TM V7 are on the left-hand side. Colours: Atoms: cyan – carbon, red – oxygen, blue – nitrogen, white – hydrogen; Structures: blue – α-helix, cyan – coil; green – turn; Interactions: yellow – hydrogen bonds. NMS makes hydrogen bonds with Q179 by its oxygen in azatricycle group, and with E172 by its hydroxy group.

Supplementary Video Legends

Supplementary Video 1: Molecular dynamics of steered dissociation of QNB from the wild type M_2 receptor

Video of sample trajectory of molecular dynamics of steered dissociation of QNB from the wild type M_2 receptor. Rate: 250 ps per frame, 6.25 ns per second. Length 200 ns.

Supplementary Video 2: Molecular dynamics of steered dissociation of NMS from the wild type M_2 receptor

Video of sample trajectory of molecular dynamics of steered dissociation of NMS from the wild type M_2 receptor. Rate: 250 ps per frame, 6.25 ns per second. Length 200 ns.

Supplementary Video 3: Molecular dynamics of steered dissociation of QNB from the D97N mutant of M_2 receptor

Video of sample trajectory of molecular dynamics of steered dissociation of QNB from the D97N mutant of M_2 receptor. Rate: 250 ps per frame, 6.25 ns per second. Length 200 ns.

Supplementary Video 4: Molecular dynamics of steered dissociation of NMS from the D97N mutant of M_2 receptor

Video of sample trajectory of molecular dynamics of steered dissociation of NMS from the D97N mutant of M_2 receptor. Rate: 250 ps per frame, 6.25 ns per second. Length 200 ns.

Supplementary Video 5: Molecular dynamics of steered aissociation of QNB with the wild type M_2 receptor

Video of sample trajectory of molecular dynamics of steered association of QNB with the wild type M_2 receptor. Rate: 125 ps per frame, 3.125 ns per second. Length 10.3 ns.

Supplementary Video 6: Molecular dynamics of steered association of NMS with the wild type M_2 receptor

Video of sample trajectory of molecular dynamics of steered association of NMS with the wild type M_2 receptor. Rate: 125 ps per frame, 3.125 ns per second. Length 11.5 ns.

Supplementary Video 7: Molecular dynamics of steered association of QNB with the D97N mutant of M_2 receptor

Video of sample trajectory of molecular dynamics of steered association of QNB with the D97N mutant of M_2 receptor. Rate: 125 ps per frame, 3.125 ns per second. Length 9.0 ns.

Supplementary Video 8: Molecular dynamics of steered association of NMS with the D97N mutant of M_2 receptor

Video of sample trajectory of molecular dynamics of steered association of QNB with the D97N mutant of M_2 receptor. Rate: 125 ps per frame, 3.125 ns per second. Length 8.7 ns.
